# Desialylation is a mechanism of Fc-independent platelet clearance and a therapeutic target in immune thrombocytopenia

**DOI:** 10.1038/ncomms8737

**Published:** 2015-07-17

**Authors:** June Li, Dianne E. van der Wal, Guangheng Zhu, Miao Xu, Issaka Yougbare, Li Ma, Brian Vadasz, Naadiya Carrim, Renata Grozovsky, Min Ruan, Lingyan Zhu, Qingshu Zeng, Lili Tao, Zhi-min Zhai, Jun Peng, Ming Hou, Valery Leytin, John Freedman, Karin M. Hoffmeister, Heyu Ni

**Affiliations:** 1 Department of Laboratory Medicine and Pathobiology, University of Toronto, Toronto, Ontario, Canada M5S 1A8; 2 Toronto Platelet Immunobiology Group, Toronto, Ontario, Canada M5B 1W8; 3 Department of Laboratory Medicine, Keenan Research Centre for Biomedical Science of St. Michael's Hospital, Toronto, Ontario, Canada M5B 1W8; 4 Canadian Blood Services, Ottawa, Ontario, Canada K1G 4J5; 5 Translational Medicine Division, Brigham and Women's Hospital, Harvard Medical School, Boston, Massachusetts 02115, USA; 6 Department of Hematology, Anhui Medical University, Hefei 230032, China; 7 Department of Hematology, Qilu Hospital, Shandong University, Jinan 250012, China; 8 Department of Medicine, University of Toronto, Ontario, Canada M5S 1A8; 9 Department of Physiology, University of Toronto, Ontario, Canada M5S 1A8

## Abstract

Immune thrombocytopenia (ITP) is a common bleeding disorder caused primarily by autoantibodies against platelet GPIIbIIIa and/or the GPIb complex. Current theory suggests that antibody-mediated platelet destruction occurs in the spleen, via macrophages through Fc–FcγR interactions. However, we and others have demonstrated that anti-GPIbα (but not GPIIbIIIa)-mediated ITP is often refractory to therapies targeting FcγR pathways. Here, we generate mouse anti-mouse monoclonal antibodies (mAbs) that recognize GPIbα and GPIIbIIIa of different species. Utilizing these unique mAbs and human ITP plasma, we find that anti-GPIbα, but not anti-GPIIbIIIa antibodies, induces Fc-independent platelet activation, sialidase neuraminidase-1 translocation and desialylation. This leads to platelet clearance in the liver via hepatocyte Ashwell–Morell receptors, which is fundamentally different from the classical Fc–FcγR-dependent macrophage phagocytosis. Importantly, sialidase inhibitors ameliorate anti-GPIbα-mediated thrombocytopenia in mice. These findings shed light on Fc-independent cytopenias, designating desialylation as a potential diagnostic biomarker and therapeutic target in the treatment of refractory ITP.

## Abstract (web summary)

Immune thrombocytopenia (ITP) is caused by autoantibody-mediated platelet clearance, but refractoriness to current immunomodulatory therapies is common. Here the authors show that desialylated platelets can be cleared via hepatic Ashwell–Morell receptor, a process that can be attenuated by sialidase inhibitors, suggesting a new therapy for ITP.

## Graphical abstract

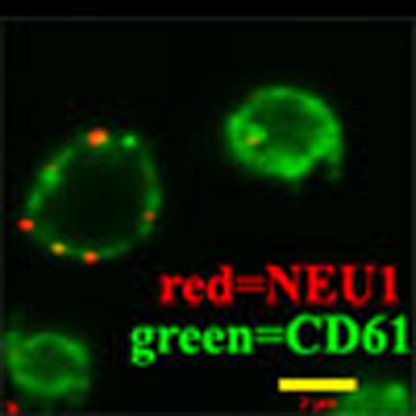

Immune thrombocytopenia (ITP) is a common bleeding disorder characterized by increased destruction of autologous platelets^1^,^2^,^3^. Low platelet counts increase the risk for bleeding, which leads to severe intracranial haemorrhage in ∼5% of patients^1^,^2^,^3^. ITP patients live with the risk of fatal bleeding and many undergo long-term therapeutic regimens to manage platelet counts, and suffer a marked decrease in quality of life^4^. First-line treatments include immunosuppressive and immunomodulatory agents (that is, corticosteroids, intravenous immunoglobulin G (IVIG) and anti-RhD therapy). Splenectomy has to be considered for patients with a persistent lack of response to treatment^5^. However, it is estimated that 15–25% of patients are inexplicably refractory to first-line therapies and even splenectomy^6^. To date, there is no reliable measurement in the clinical setting to predict the success or failure of any ITP treatment^5^,^7^.

Autoantibodies targeting platelet surface glycoprotein(s) (GP) have been demonstrated to be the major factors responsible for platelet clearance^2^,^8^,^9^. Approximately 70–80% of patients have autoantibodies against GPIIbIIIa (integrin αIIbβ3), 20–40% against the GPIb complex and some patients have autoantibodies against both or other GPs^11^,^12^,^13^. Platelet destruction following autoantibody binding has generally been considered to occur in the spleen, through binding of the Fc portion of immunoglobulins on the platelet surface to FcγRIIa and FcγRIIIa on tissue macrophages of the reticuloendothelial system^2^. Accordingly, first-line therapies, such as IVIG and anti-Rh(D), target these Fc- and FcγR-dependent mechanisms to restore platelet numbers^10^. Unexpectedly, we and others have identified a novel mechanism of Fc-independent thrombocytopenia, in which antibodies against GPIbα, but not those against GPIIbIIIa, can induce thrombocytopenia via their F(ab)_2_ (Fc independent) and in *FcγR*^*−/−*^ mice^11^,^12^. We further reported that most anti-GPIbα antibody-mediated thrombocytopenia is resistant to IVIG treatment^12^. This is consistent with subsequent reports in humans, including our recent large patient cohort study^13^,^14^,^15^. In addition, our retrospective studies suggest that ITP patients with anti-GPIbα antibodies are also more likely to be refractory to steroid treatments^16^. These data indicate that anti-GPIbα antibodies are able to uniquely induce platelet clearance in an Fc-independent manner in murine models, which may also be true in human ITP. However, the nature of this novel Fc-independent mechanism of platelet clearance is unknown.

GPIIbIIIa and the GPIb complex are structurally and functionally distinct platelet receptors. Although different outside-in signalling pathways have been observed between these two receptors following ligand stimulation^17^,^18^, the downstream effects of autoantibody binding have not been adequately studied. Thus, possible differences in pathogenesis and therapy between anti-GPIIbIIIa- and anti-GPIb-mediated ITP remain to be elucidated.

As the second-most abundant platelet surface receptor, GPIbα is the largest subunit and possesses all known extracellular ligand-binding sites of the GPIb complex (that is, GPIb-IX-V). Binding of GPIbα to the von Willebrand factor initiates GPIbα outside-in signalling, which can subsequently activate GPIIbIIIa leading to platelet aggregation^17^,^19^. GPIbα is also the most heavily glycosylated platelet surface protein with ∼60% carbohydrate by weight^20^. It contains both *N*- and *O*-linked carbohydrate chains capped with sialic acid, contributing to 64% of the total sialic acid content of the platelet^21^. Deglycosylation of GPIbα was reported to result in hepatic clearance of cold-stored platelets following transfusion^22^,^23^,^24^. This suggests that glycan modification of GPIbα may be sufficient to induce platelet clearance, likely via an Fc-independent pathway. We previously found that antisera against GPIbα can induce platelet activation^25^; however, other downstream platelet responses following antibody binding have not been adequately studied. Whether antibody binding may also lead to deglycosylation and contribute to platelet clearance, and whether this may account for the differences in pathogenesis and response to therapy in ITP has not been investigated.

In this study, by utilizing unique anti-GPIbα and anti-GPIIbIIIa monoclonal antibodies (mAbs) as well as human ITP plasma samples, we show that significant platelet activation and desialylation occurs predominantly in the presence of anti-GPIbα, but not anti-GPIIbIIIa antibodies *in vitro* and *in vivo*. Furthermore, anti-GPIbα-mediated desialylation of platelets led to FcγR-independent clearance via hepatocytes. Most importantly, the use of sialidase inhibitors mitigated thrombocytopenia in a murine model of anti-GPIbα-mediated ITP. Thus, we identify a mechanism of platelet clearance previously undescribed. In addition, we introduce a new diagnostic method to detect platelet desialylation in ITP and establish sialidase inhibitors as a potential therapeutic for refractory ITP.

## Results

### Generation of novel anti-GPIbα and anti-GPIIbIIIa antibodies

To examine the effects of anti-GPIbα and anti-GPIIbIIIa antibodies on platelet function and clearance, we utilized gene-deficient (^*−/−*^) mice to generate a panel of mouse anti-mouse-GPIbα and -GPIIbIIIa mAbs, which possess cross-reactivity against other species, including tested human, rat and/or pig antigens (Table 1). These are the first syngeneic anti-GPIbα antibodies utilized in the study of ITP, thus circumventing xenogeneic antibody complications. To our knowledge, this is also the first report that anti-GPIbα antibodies can cross-react with GPIbα from different species. Further characterization of these antibodies revealed that some of these mAbs affect ristocetin/botrocetin- or ADP/thrombin-induced platelet aggregation, which are mediated by GPIbα^26^ or GPIIbIIIa, respectively (Table 1)^27^,^28^,^29^. Injection of these antibodies into mice induced significant platelet decrease (thrombocytopenia) with no significant effects on red or white blood cell counts (Supplementary Table 1). Thus, these mAbs may serve useful in the study of different animal models of human diseases, including thrombosis and hemostasis, as well as other immune-mediated thrombocytopenias.

### Anti-GPIbα antibodies induce significant platelet activation

It has been previously reported that antibodies against GPIbα may induce platelet activation^25^,^30^,^31^. Therefore, we investigated whether our newly developed anti-GPIbα mAbs may also affect platelet function. We incubated murine and human platelets with various anti-GPIbα or anti-GPIIbIIIa mAbs of different immunoglobulin (Ig)G subclasses (Table 1). Three anti-GPIbα mAbs (NIT A, NIT B and NIT F) were employed in our human platelet studies as they were, to the best of our knowledge, the first and only currently available mAbs that are cross-reactive to human GPIbα.

**Figure 1:**
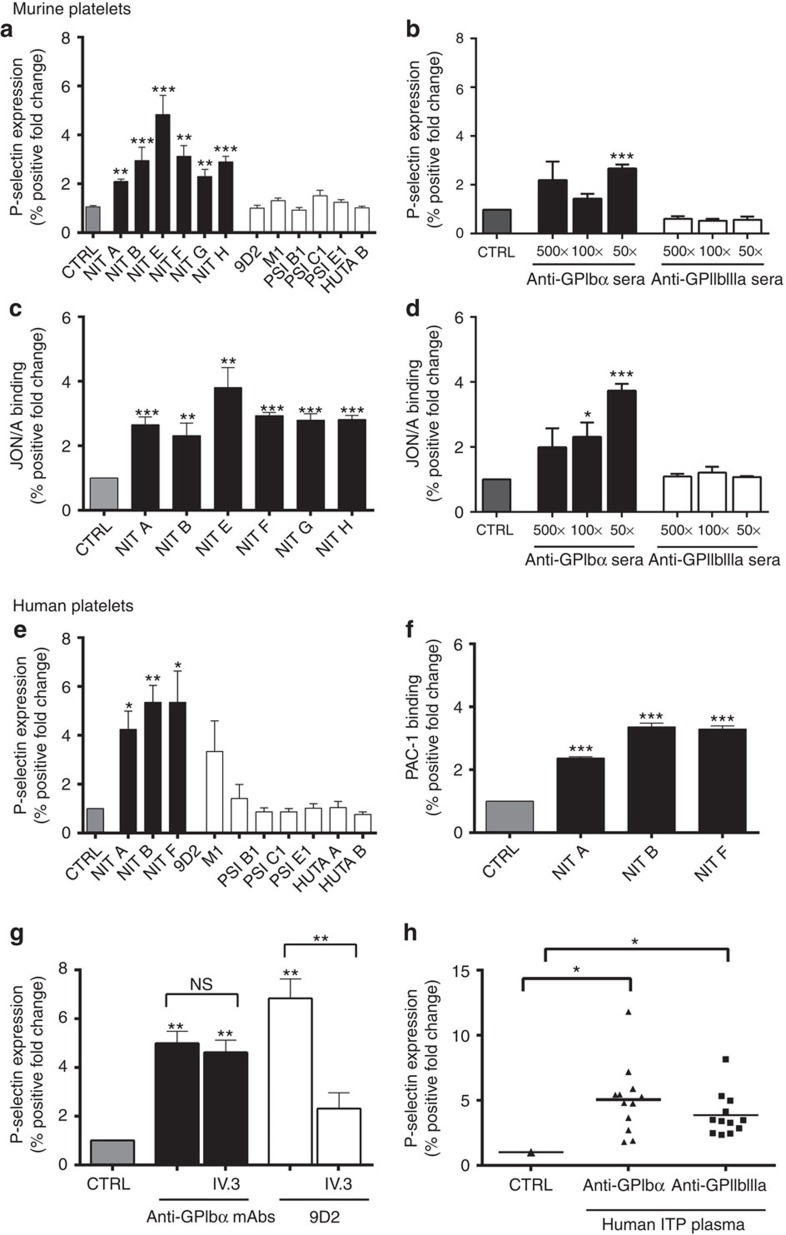
Anti-GPIbα antibodies induce platelet activation. Surface P-selectin expression on murine (**a**,**b**) or human platelets (**e**,**g**,**h**) and JON/A binding on murine (**c**,**d**) or PAC-1 binding on human platelets (**f**) were measured by flow cytometry following incubation with mAbs (**a**,**c**,**e**,**f**,**g**) or antisera (**b**,**d**). (**g**) FcγRIIa/III blocker IV.3 was incubated with mAb 9D2 and platelets, and platelet P-selectin was assessed following. Only the healthy donor platelets that were significantly activated in the presence of 9D2 were tested; *n*=3. (**h**) P-selectin expression was measured in healthy human platelets following incubation with anti-GPIbα (ITP-1 to ITP-12) or anti-GPIIbIIIa (ITP-a to ITP-l) antibody-positive ITP patient plasma. All flow cytometry data are expressed as fold change from nonspecific murine IgG (murine)- or IVIG (human)-treated control platelets (CTRL). Anti-GPIbα mAbs shown as mean±s.e.m. of individual mAbs. *P<0.05, **P<0.01, ***P<0.001 versus CTRL as analysed by the Student's *t*-test (**a**–**f**) or one-way analysis of variance followed by Bonferroni *post ho*c analysis (**g**,**h**). NS, not significant.

Platelet granule secretion (surface expression of P-selectin) was significantly increased in the presence of all anti-GPIbα mAbs tested in both mouse and human platelets (Fig. 1a,e). To circumvent biases stemming from the specific binding epitopes of individual antibody clones, we also tested polyclonal sera against GPIbα and GPIIbIIIa (generated in knockout mice immunized with wild-type (WT) platelets^25^). Similarly, we found anti-GPIbα, but not anti-GPIIbIIIa, sera induced platelet P-selectin expression dose dependently (Fig. 1b). Consistent with P-selectin expression, we detected significantly increased platelet GPIIbIIIa activation following anti-GPIbα treatment, as measured by JON/A (Fig. 1c,d) and PAC-1 binding (Fig. 1f). JON/A and PAC-1 are specific mAbs against murine and human GPIIbIIIa, respectively, that bind exclusively to the cation-dependent high-affinity conformation of GPIIbIIIa^32^,^33^. Anti-GPIbα-induced platelet aggregation was also visualized by light microscopy (Supplementary Fig. 1). None of the anti-GPIIbIIIa antibodies tested induced detectable increases in murine platelet P-selectin expression (Fig. 1a,b). However, some anti-GPIIbIIIa antibodies, in particular mAb clone 9D2, did induce significant P-selectin expression on human platelets of certain (3 of the 10) healthy donors (Fig. 1e). We suspected this may have been due to immune-complex binding of FcγRIIa expressed exclusively on human but not murine platelets^34^, which has been previously shown to cause platelet granule release independent of additional agonists^35^. To test this possibility, we utilized the FcγRIIa/III blocker IV.3, and found that it completely attenuated 9D2-mediated P-selectin expression. Interestingly, IV.3 did not have any significant effect on any anti-GPIbα mAb-mediated human platelet activation (Fig. 1g).

Finally, utilizing plasma samples obtained from a cohort of ITP patients, we found anti-GPIbα ITP plasma induced significant P-selectin expression, while anti-GPIIbIIIa ITP plasma induced only moderate P-selectin expression (Fig. 1h). These experiments demonstrate that antibodies against GPIbα induced higher platelet activation, while anti-GPIIbIIIa antibodies may induce platelet activation in some human platelets through an FcγRIIa-dependent mechanism (Fig. 1g). These differences between anti-GPIbα and anti-GPIIbIIIa antibody-induced platelet activation may serve as the basis for the mechanistic differences between anti-GPIIbIIIa Fc-dependent and anti-GPIbα Fc-independent platelet clearance.

### Anti-GPIbα antibodies induce platelet desialylation

**Figure 2:**
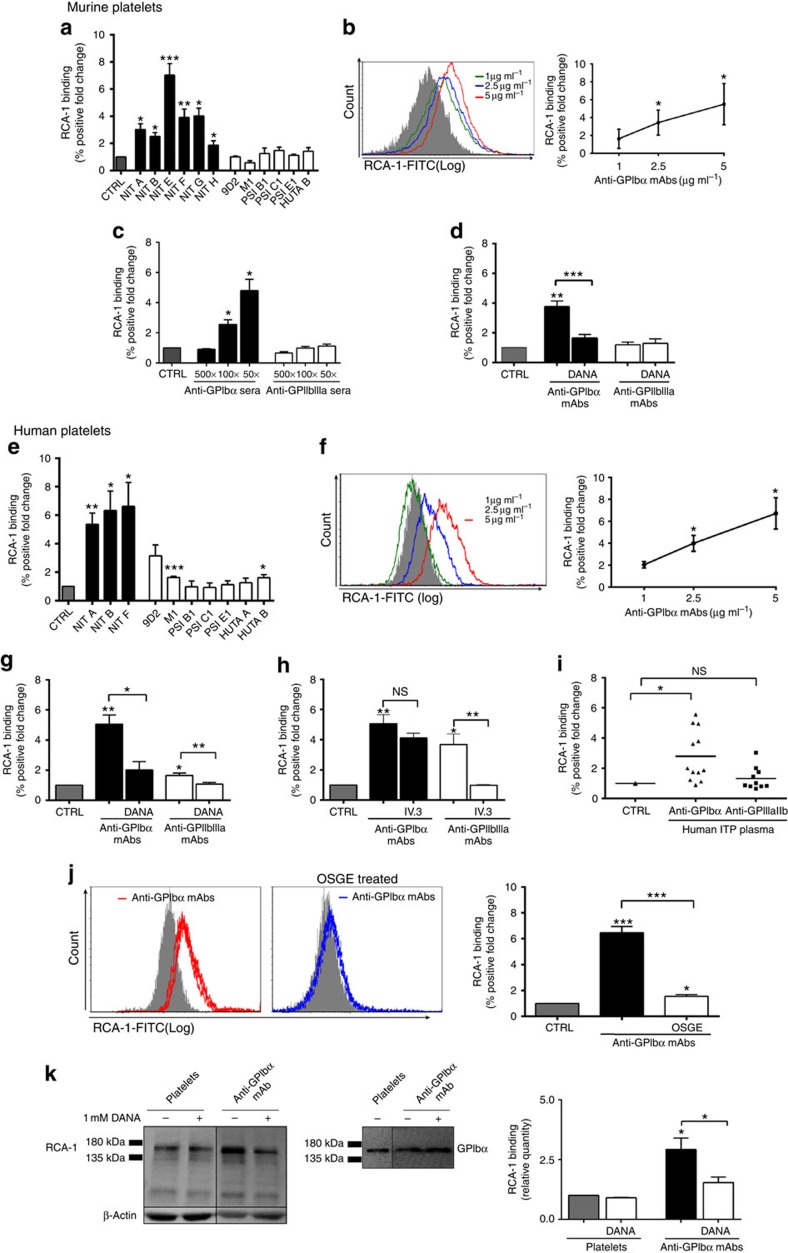
Antibody-mediated platelet desialylation occurs mainly on the GPIbα subunit. Galactose exposure was detected with RCA-1 binding and measured by flow cytometry in murine (**a**–**d**) and human (**e**–**j**) platelets following incubation with mAbs (**a**,**b**,**d**,**e**,**f**–**h**,**j**,**k**) or antisera (**c**) *n*=10–20. (**d**,**g**) Co-incubation with sialidase inhibitor DANA prior to addition of mAbs to murine (**d**) or human (**g**) platelets; *n*=8. (**h**) FcγRII/III blocker IV.3 was incubated with mAbs (9D2, M1 or HUTA B) and platelets, RCA-1 binding was assessed following. Only the healthy donor platelets that were significantly desialylated in the presence of these mAbs were tested; *n*=4. (**i**) RCA-1 binding was measured in healthy human platelets following incubation with anti-GPIbα (ITP-1 to ITP-12) or anti-GPIIbIIIa (ITP-a to ITP-l) antibody-positive ITP patient plasma. (**j**) anti-GPIbα-mediated RCA-1 binding was assessed following removal of GPIbα with OSGE. (**k**) Representative western blot of RCA-1 binding (left), and probing with commercial anti-GPIbα antibody (right) to confirm the identity of the RCA-1-positive band following incubation with anti-GPIbα mAb (NIT F). RCA-1 binding on GPIbα was also quantified by protein densitometry in the presence of DANA. All flow cytometry data are expressed as fold change from nonspecific murine IgG (murine)- or IVIG (human)-treated control platelets (CTRL). Anti-GPIbα mAbs shown as mean±s.e.m. of individual mAbs. *P<0.05, **P<0.01, ***P<0.001 versus CTRL as analysed by the Student's *t*-test (**a**–**c**,**e**,**f**) or one-way analysis of variance followed by Bonferroni *post hoc* (**d**,**g**–**k**). NS, not significant.

Antibody-mediated platelet desialylation has not been previously investigated. To test whether anti-GPIbα antibodies also induce platelet desialylation, we quantified the binding of fluorescein-conjugated *Ricinus communis* agglutinin I (RCA-1) lectins, which specifically target exposed galactose residues following GP desialylation^23^,^36^. We found that all anti-GPIbα mAb-treated platelets (both murine and human) exhibited significant desialylation (Fig. 2a,e). This desialylation was dose dependent, with increasing concentrations of both our mAb (Fig. 2b,f) and GPIbα antisera (Fig. 2c). Platelet glycosylation changes were further characterized with additional lectins including peanut agglutinin (PNA), Sambucus Nigra Lectin and Maackia Amurensis Lectin II. Although only significantly increased binding of PNA was observed on murine platelets, increased binding of all the lectins to human platelets occurred following anti-GPIbα mAbs incubations (Supplementary Fig. 2). To confirm that desialylation is a direct consequence of antibody binding, we measured anti-GPIbα-mediated desialylation in the presence of 2-deoxy-2,3-didehydro-*N*-acetylneuraminic acid (DANA), a synthetic mammalian and bacterial/viral pan-sialidase inhibitor, which possesses a reported half-maximal inhibitory concentration of ∼80 μM against mammalian neuraminidase-1 (NEU1)^37^. DANA was found to significantly decrease anti-GPIbα-mediated desialylation of murine and human platelets (Fig. 2d,g). In addition, DANA had no effect on desialylation of *GPIbα*^*−/−*^ platelets treated with anti-GPIbα mAbs (Supplementary Fig. 3).

In contrast, although anti-GPIIbIIIa mAbs did not significantly affect desialylation on murine platelets, clones 9D2, M1 and HUTA B did cause desialylation on platelets from certain individual donors (Fig. 2e). Similar to what was observed in platelet activation, desialylation caused by anti-GPIIbIIIa mAbs (9D2, M1 and HUTA B) was FcγRIIa dependent, as blocking with IV.3 completely attenuated the response (Fig. 2h). Importantly, when healthy human platelets were incubated with plasma from ITP patients, anti-GPIbα ITP plasma triggered significant RCA-1 binding, while anti-GPIIbIIIa ITP plasma-induced RCA-1 binding was moderate (Fig. 2i).

Together these results confirm that increased sialidase activity resulting in desialylation directly follows from anti-GPIbα antibody binding. Thus, platelet desialylation may be an important biomarker for platelet activation and for anti-GPIb-mediated ITP.

### The GPIbα subunit is predominantly desialylated

Given that GPIbα is the most heavily glycosylated subunit on the platelet surface^38^, we suspected that GPIbα may be the principle target of desialylation. To test this, we utilized *O*-sialoglycoprotein endopeptidase (OSGE) to selectively remove the extracellular N terminus of GPIbα^39^. Evaluation of RCA-1 binding before and after OSGE treatment revealed that binding decreased to almost baseline levels following removal of GPIbα (Fig. 2j). In addition, following anti-GPIbα mAb treatment, platelet lysate was blotted with RCA-1 and a commercial anti-GPIbα antibody that identified a polypeptide corresponding to around 150 kDa. Western blotting confirmed RCA-1 binding was strongest on GPIbα and was susceptible to DANA inhibition (Fig. 2k). These data indicate that GPIbα is likely the dominant target of anti-GPIbα-mediated desialylation.

### Anti-GPIbα antibodies induce surface expression of NEU1

**Figure 3:**
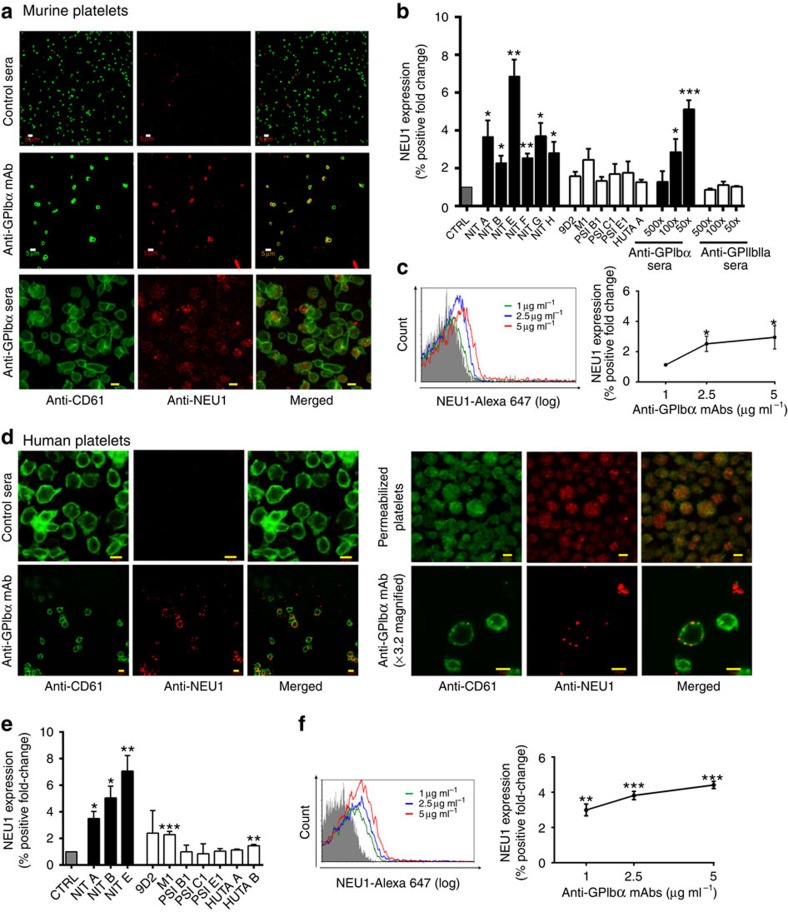
Anti-GPIbα antibodies induce surface expression of NEU1. Representative confocal images of surface expression of NEU1 on murine (**a**) and human (**d**) platelets stained with anti-NEU1 and anti-CD61 following incubations with anti-GPIbα mAb (NIT G (murine), NIT B (human)) or anti-GPIbα sera (murine). All other mAbs were also tested with similar results; *n*=5–8. Total NEU1 was detected in permeabilized human platelets; *n*=2. White scale bars, 5 μM; yellow scale bars, 2 μM. (**b**,**c**,**e**,**f**) Flow cytometric analysis of surface NEU1 expression on murine (**b**,**c**) and human (**e**,**f**) platelets following incubations with anti-GPIbα mAb or sera. Anti-GPIbα mAbs shown as mean±s.e.m. of individual mAbs; *n*=5–8. All flow cytometry data are expressed as fold change from nonspecific murine IgG (murine)- or IVIG (human)-treated control platelets (CTRL). Anti-GPIbα mAbs shown as mean±s.e.m. of individual mAbs. *P<0.05, **P<0.01, ***P<0.001 versus CTRL as analysed by the Student's *t*-test.

Since sialidase NEU1 has been reported to be involved in platelet desialylation^40^, we established a method to detect surface platelet NEU1 using flow cytometry, and found that anti-GPIbα, but not anti-GPIIbIIIa or isotype control antibodies, induced NEU1 translocation onto both murine and human non-permeabilized platelets (Fig. 3a–f; Supplementary Fig. 4). This NEU1 was found to be abundant in the granules of permeabilized platelets (Fig. 3d). Consistent with our RCA-1-binding results (Fig. 2b,f), we observed a dose-dependent increase of NEU1 translocation with increasing concentrations of anti-GPIbα mAb (Fig. 3c,f). Triple staining of P-selectin, RCA-1 and NEU1 on platelets following anti-GPIbα mAb incubations revealed significant co-expression and correlation between these markers within the same platelet population (*P*<0.001 as assessed by Pearson's correlation, Supplementary Figs 5 and 6). These data not only strongly implicate NEU1 as the enzyme mediating desialylation, but also link desialylation with platelet activation.

### Platelet activation and desialylation positive feedback loop

**Figure 4:**
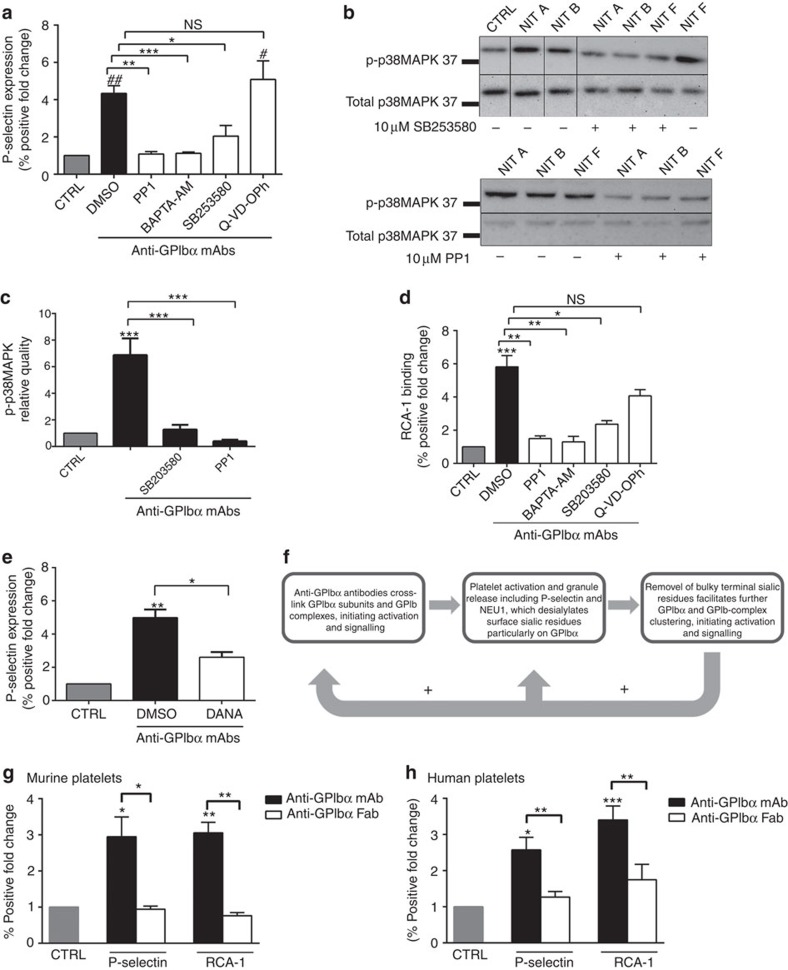
Anti-GPIbα platelet activation and desialylation is a positive feedback loop. (**a**,**d**) Human platelets were pre-incubated with inhibitors of platelet activation including intracellular Ca^2+^ flux (BAPTA-AM), phosphorylation of P38MAPK (SB203580) and Src kinase (PP1) prior to addition of anti-GPIbα mAbs. Following which, platelet activation (P-selectin expression) (**a**) or desialylation (RCA-1 binding) (**d**) was detected via flow cytometry. # and ## indicate comparison with CTRL for **a** only; *n=*8. (**b**) Representative western blots of whole-platelet lysate following incubations with GPIbα mAbs with or without indicated inhibitors. Membranes were probed for phosphorylated p38MAPK (p-p38MAPK) then stripped and re-probed for total pMAP38K. (**c**) Densitometry protein quantification of p-P38MAPK detected in **b**. Data representative of three separate experiments. (**e**) Sialidase inhibitor DANA was added prior to anti-GPIbα mAb incubation. DANA-mediated inhibition of antibody-induced platelet activation was measured by P-selectin expression and detected via flow cytometry. *n=*6. (**f**) Schematic flow chart illustrating platelet activation–desialylation-positive feedback loop. Following initial anti-GPIbα antibody crosslinking of GPIbα subunits, activation signalling occurs leading to surface translocation of NEU1. NEU1 cleavage of terminal sialic residues on a GPIbα subunit facilitates receptor clustering, resulting in amplification of platelet activation. (**g**,**h**) NIT A, NIT B and NIT F Fab fragments were generated and their effects on platelet activation and desialylation on murine (**g**) and human platelets (**h**) were analysed by flow cytometry. *n*=5. All flow cytometry data are expressed as fold change from nonspecific murine IgG (murine)- or IVIG (human)-treated control platelets (CTRL), unless otherwise indicated. Anti-GPIbα mAbs shown as mean±s.e.m. of individual mAbs. *^,#^*P*<0.05, **^,##^*P*<0.01, ***P<0.001 as assessed by one-way analysis of variance followed by Bonferroni *post hoc* analysis. NS, not significant.

We next determined whether the observed anti-GPIbα antibody effects were acting through canonical GPIbα activation signalling cascades. We first tested whether various inhibitors of known downstream GPIbα activation signalling molecules could decrease anti-GPIbα mAb-mediated platelet activation. We found inhibitors of intracellular Ca^2+^ flux (BAPTA-AM), P38MAPK activity (SB203580) and Src kinases (PP1) all significantly decreased anti-GPIbα antibody-induced human platelet activation as measured by P-selectin, while Q-VD-OPh, a pan caspase inhibitor of platelet apoptosis did not appear to have any effect (Fig. 4a). Western blot analysis further demonstrated anti-GPIbα mAb can directly mediate GPIbα downstream signalling as evidenced by increased phosphorylation of p38MAPK (Fig. 4b), which could be inhibited by SB203580 and PP1 as quantified by densitometry (Fig. 4b,c). These data indicate that anti-GPIbα antibodies directly mediate platelet activation through endogenous GPIbα signalling pathways.

To further elucidate the link between anti-GPIbα-mediated platelet activation and desialylation, we first tested whether inhibitors of platelet activation may affect platelet desialylation. We found inhibitors of GPIbα activation SB203580, BAPTA-AM and PP1, but not platelet apoptosis (Q-VD-OPh), markedly inhibited anti-GPIbα mAb-induced desialylation (Fig. 4d). This indicates that GPIbα activation is a prerequisite for anti-GPIbα mAb-mediated desialylation. Conversely, to test whether desialylation had any effect on platelet activation, we treated platelets with the sialidase inhibitor DANA in the presence of anti-GPIbα mAbs. DANA strongly inhibited anti-GPIbα-induced P-selectin expression (Fig. 4e). Together, these data establish that, while GPIbα activation may be required for desialylation, desialylation itself may further potentiate platelet activation. It has been previously reported that removal of bulky terminal sialic residues can facilitate GPIb-receptor clustering and outside-in signalling^41^,^42^. Thus, anti-GPIbα antibody binding, which initiates platelet activation, NEU1 translocation and GPIbα desialylation, creates a positive feedback loop culminating in additional P-selectin expression and platelet desialylation (Fig. 4f).

Since GPIbα clustering has been linked to platelet signalling and activation^43^,^44^, we generated Fab monovalent antibody fragments from several anti-GPIbα mAbs. Fab fragments incubated with both human and murine platelets did not cause significant platelet activation or desialylation (Fig. 4g,h). We thus propose that GPIbα antibody crosslinking is fundamental for antibody-mediated platelet activation and desialylation.

### Platelet activation and desialylation occur *in vivo*

**Figure 5:**
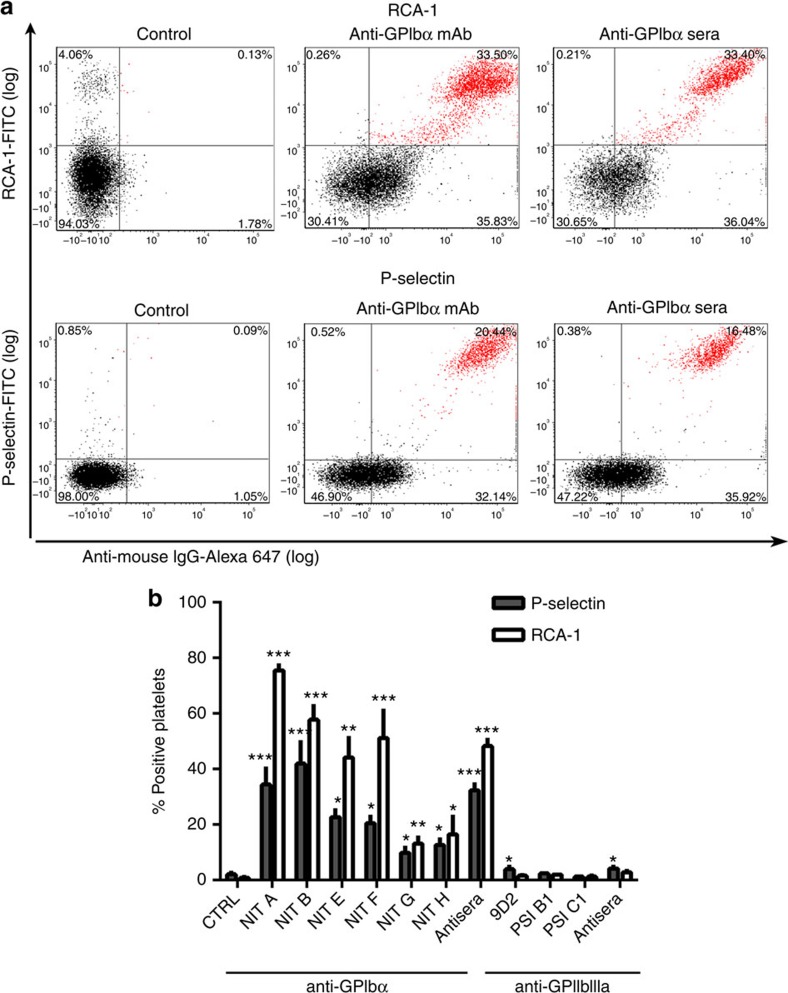
Anti-GPIbα mAbs induce platelet activation and desialylation *in vivo*. (**a**,**b**) ITP was induced by intraperitoneal injection of anti-GPIbα mAbs (NIT A, NIT B, NIT E, NIT F, NIT G and NIT H) or sera. At 16 h post injection, platelets isolated from the mice were examined for activation (P-selectin) and desialylation (RCA-1) by flow cytometry; *n*=3 per mAb. (**a**) Representative dot plots of isolated washed platelets from anti-GPIbα mAb (NIT E)- and sera-injected mice double-stained for anti-mouse IgG (against anti-GPIbα antibodies) (*x* axis) and RCA-1/P-selectin (*y* axis).

To test whether our *in vitro* observations occur *in vivo*, anti-GPIbα and anti-GPIIbIIIa mAbs or antisera were injected into female BALB/c mice to induce thrombocytopenia. Platelets isolated from this model 16 h post injection were co-stained for anti-mouse IgG and RCA-1 or P-selectin to assess the percentage of anti-GPIbα antibody-bound platelets that were positive for platelet activation and desialylation. We found that in NIT A-, NIT B- and NIT F-injected mice, >50% of antibody-bound platelets were desialylated, while NIT E-, NIT G- and NIT H-injected mice ranged from 20 to 40%. In addition, there was a significant proportion of antibody-bound platelets positive for P-selectin expression, albeit at lower percentages than desialylation (Fig. 5a,b). In contrast, platelets from thrombocytopenic mice injected with anti-GPIIbIIIa mAb or antisera did not exhibit these effects (Fig. 5b). Overall, *in vivo* antibody-mediated platelet activation and desialylation were observed to be more pronounced than *in vitro*. Thus, anti-GPIbα antibodies induced significant increases in platelet activation and desialylation in a clinically relevant model of ITP.

### Anti-GPIbα-opsonized platelets can be cleared via the AMR

**Figure 6:**
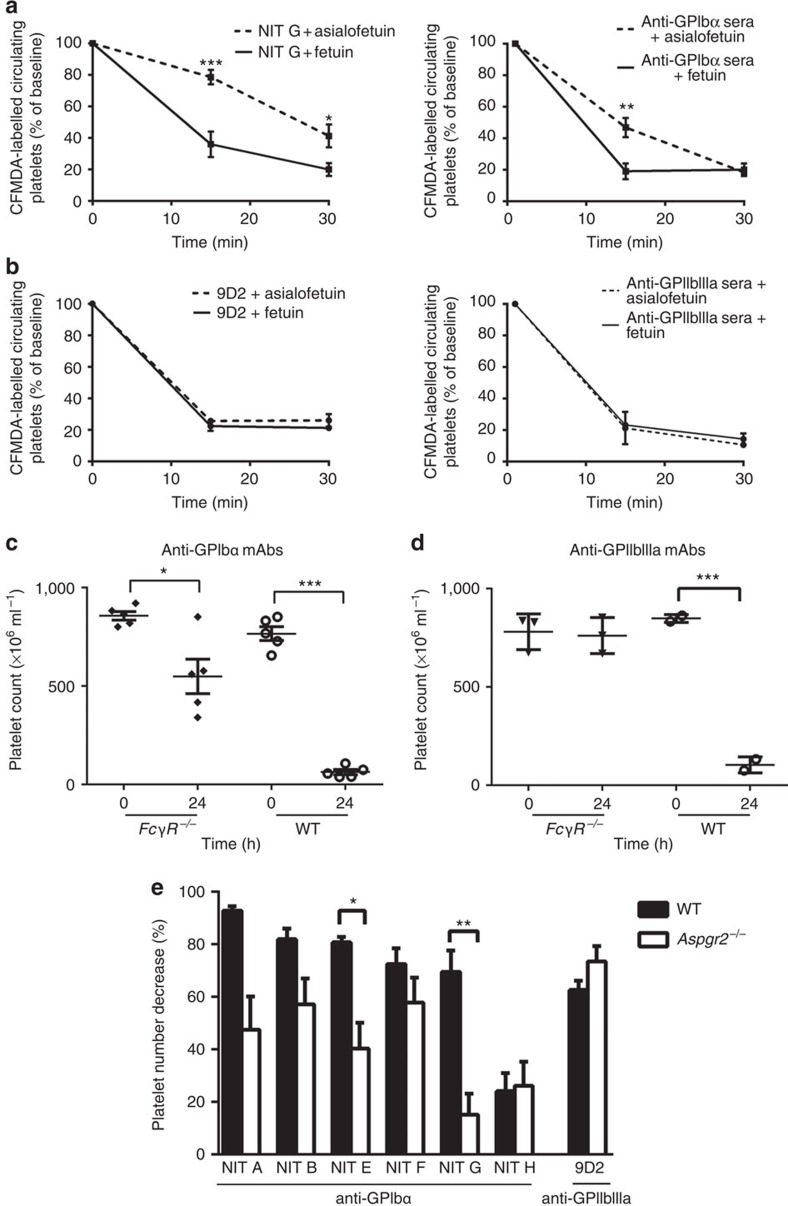
Fc-independent anti-GPIbα-mediated thrombocytopenia occurs via the AMR. Mice were co-injected with (**a**) anti-GPIbα (NIT G) or (**b**) anti-GPIIbIIIa mAb (9D2)- or polyclonal sera-opsonized CFMDA-labelled platelets with asialofetuin (AMR inhibitor) or fetuin (a nonspecific control). Mice were bled at indicated time points and the percentage of fluorescently labelled platelets remaining in circulation was assessed with flow cytometry; *n*=6. *P<0.05 versus Fetuin-injected group at same time point as analysed by two-way analysis of variance followed by Bonferroni *post hoc* analysis. (**c**–**e**) Same doses of indicated mAb or anti-GPIbα or anti-GPIIbIIIa mAbs (equal ratio mixture of individual antibody clones) were injected into age-matched *FcγR*^*−/−*^ (**c**,**d**) or *Aspgr*^*−/−*^ (**e**) or WT control mice. Platelet counts were taken immediately prior to antibody injection (time 0) and 24 h later (time 24). (**e**) Data are represented as a percentage calculated from [(time 0 platelet count−time 24 platelet count)/time 0 platelet count] × 100. *P<0.05, **P<0.01, ***P<0.001 as determined by the Student's *t*-test.

The Ashwell–Morell receptor (AMR), an asialoglycoprotein counter receptor predominantly expressed on hepatocytes, was reported to mediate clearance of desialylated GPs^45^, and has been linked to maintenance of normal platelet turnover through the platelet deglycosylation state^24^,^46^. We suspected that the AMR may contribute to the destruction of the antibody-mediated desialylated platelets. To test this, we inhibited the AMR using asialofetuin and assessed the clearance of anti-GPIbα-opsonized platelets. We found that following co-injection of anti-GPIbα-opsonized platelets with asialofetuin, uptake of antibody-bound platelets through the AMR was attenuated, resulting in an increase by ∼25% of circulating anti-GPIbα-bound platelets compared with fetuin, a nonspecific control (Fig. 6a). Furthermore, asialofetuin did not rescue anti-GPIIbIIIa mAb (9D2)- or antisera-opsonized platelets (Fig. 6b).

To further confirm the contribution of the AMR in Fc-independent anti-GPIbα antibody-mediated thrombocytopenia, we first injected anti-GPIbα mAbs into *FcγR*^*−/−*^ mice and found thrombocytopenia still occurred (Fig. 6c), particularly when injected with anti-GPIbα mAb NIT G (Supplementary Fig. 7a), while anti-GPIIbIIIa mAbs failed to induce a significant decrease in platelets in these mice (Fig. 6d). We next compared the severity of thrombocytopenia between Ashwell–Morell-deficient (*Aspgr2*^*−/−*^) and syngeneic WT control mice and found thrombocytopenia in *Aspgr2*^*−/−*^ mice was significantly attenuated when injected with the same concentration of anti-GPIbα mAb (Fig. 6e; Supplementary Fig. 7b), suggesting that the AMR indeed plays a significant role in the clearance of anti-GPIbα-bound platelets.

**Figure 7:**
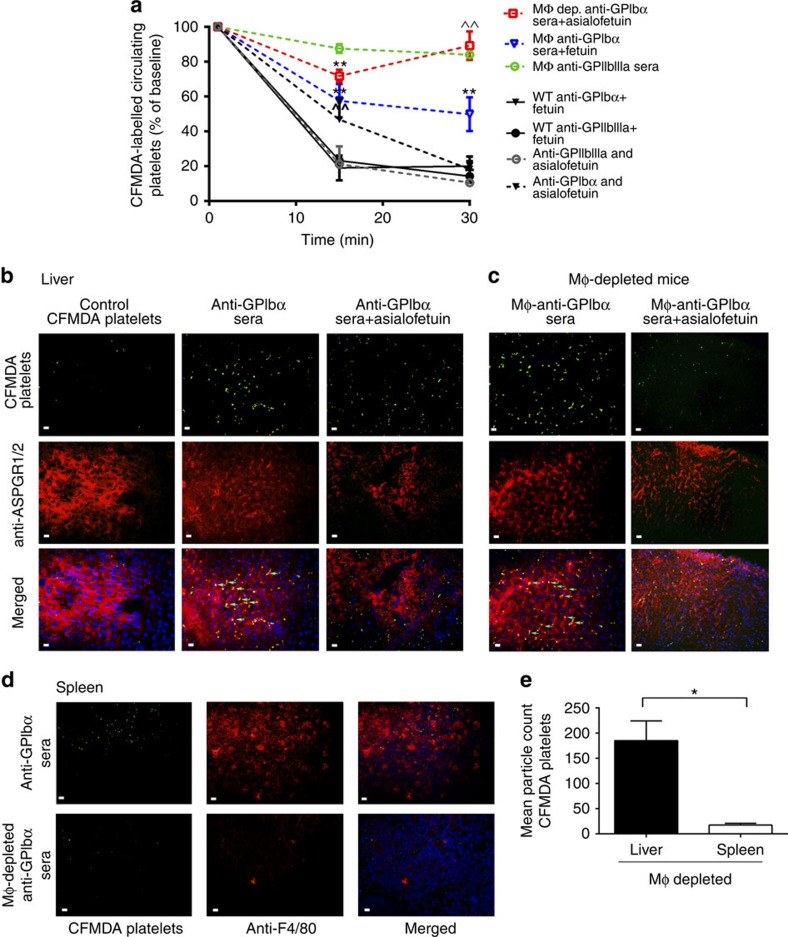
Anti-GPIbα platelet clearance in macrophage-depleted mice is via the AMR. (**a-e**) Macrophages were depleted with clondrate liposomes via intravenous injection prior to study, and CFMDA-labelled antisera-opsonized platelets were injected with asialofeuin or fetuin and circulating platelets were quantified (**a**) as described in Fig. 6. *n*=5. ^^^^*P*<0.01 versus fetuin-injected group at the same time point. *P<0.05, ^**^P<0.01 versus baseline as determined by two-way analysis of variance followed by Bonferroni *post hoc* analysis. (**b**–**d**) Tissue sections of the spleen and liver harvested from normal or macrophage-depleted mice following the above circulation studies and were stained with anti-ASPGR1/2 (liver; red) (**b**,**c**) or anti-F4/80 (spleen; red) (**d**). Nuclei were counterstained with 4′,6-diamidino-2-phenylindole (blue). Arrows indicate co-localization of CFMDA-labelled platelets with ASPGR on hepatocytes. (**e**) Fluorescent platelet (green) localization was assessed with immunofluorescent microscopy at × 60 magnification and quantified with Image J. White scale bars, 10 μM. *P<0.05 as assessed by the Student's *t*-test. Data are representative of five randomly selected fluorescent images.

The importance of the AMR in clearance of anti-GPIbα-opsonized platelets was further substantiated in macrophage-depleted mice. We found macrophage-depleted mice still maintained clearance of anti-GPIbα-opsonized platelets from circulation, while clearance of anti-GPIIbIIIa-opsonized platelets was almost completely rescued. In addition, co-injections of asialofetuin almost completely inhibited anti-GPIbα-mediated platelet clearance (Fig. 7a). This suggests that the role of the AMR in anti-GPIbα-antibody-mediated platelet clearance is dominant in the absence of macrophages.

Tissue sections of the spleen and liver revealed significant co-localization of anti-GPIbα-opsonized platelets in the liver, but not in the spleen of macrophage-depleted mice, (*P*<0.05 as determined by the Student's *t*-test, Fig. 7c–e). Treatment with asialofetuin resulted in decreased platelet localization in the livers of macrophage-depleted mice (Fig. 7c). Although asialofetuin did not decrease platelet localization in WT livers, the fluorescent platelets do not appear to co-localize with AMRs (Fig. 7b), suggesting that they may be binding to other phagocytic cells (for example, Kupffer macrophages).

These findings indicate for the first time that the AMR on hepatocytes significantly contributes to the clearance of anti-GPIbα mAb-opsonized platelets, particularly in the absence of macrophages. Thus, hepatocytes play a previously unappreciated and important role in platelet clearance in antibody-mediated thrombocytopenia.

### Sialidase inhibition can attenuate ITP

**Figure 8:**
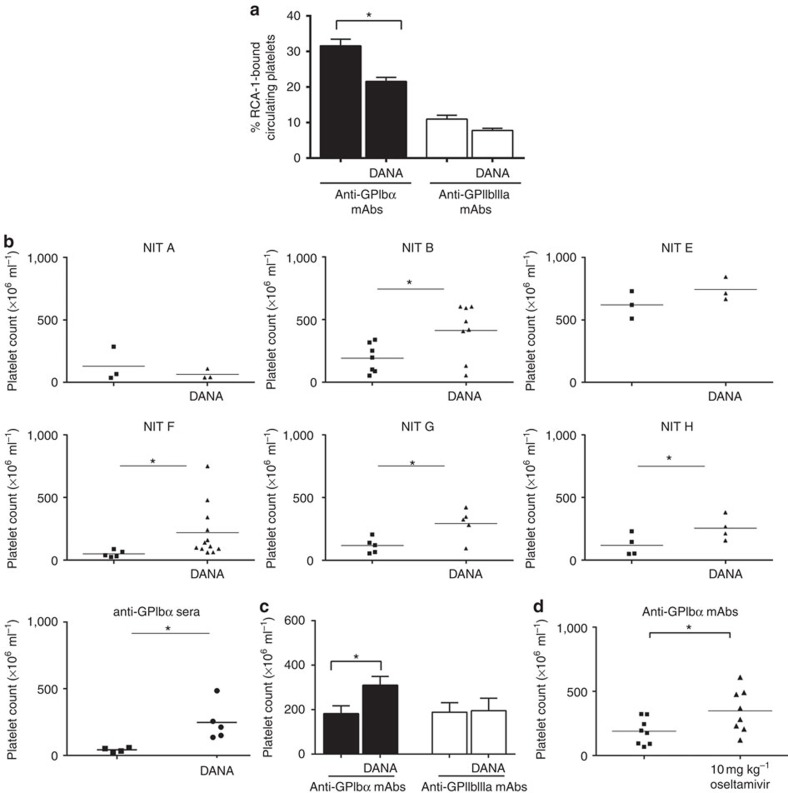
Sialidase inhibition rescues thrombocytopenia in a murine model of ITP. (**a**–**c**) DANA or PBS was injected intraperitoneally immediately prior to anti-GPIbα or anti-GPIIbIIIa antibody injection (mAb or sera) to induce thrombocyotopenia. (**a**) Platelets from antibody-injected mice were analysed for desialylation (RCA-1 binding) via flow cytometry. (**b**) Platelet numbers were enumerated and compared between DANA-treated or mock (PBS)-treated groups. (**c**) Cumulative platelet counts from all anti-GPIbα and anti-GPIIbIIIa antibody (9D2, M1, PSI C1 and antisera)-injected mice with or without DANA treatment. For individual platelet counts of anti-GPIIbIIIa-injected mice, please see Supplementary Fig. 7. (**d**) Oseltamivir phosphate was injected intravenously 2 h after anti-GPIbα mAbs injection (equal ratio mixture of individual mAb clones). Platelet enumeration was as described above. Data are shown as mean±s.e.m. platelet counts of individual mAbs. *P<0.05, ***P<0.001 as determined by the Student's *t*-test.

Patients who test positive for anti-GPIbα antibodies tend to be more refractory to standard first-line treatments, such as steroids and IVIG^13^,^14^,^16^. We explored the therapeutic potential of sialidase inhibitors in our murine ITP model. We found that pretreatment with DANA significantly decreased desialylation of platelets in anti-GPIbα, but not anti-GPIIbIIIa mAb-injected mice (Fig. 8a). Remarkably, DANA also rescued platelet counts in most of the anti-GPIbα mAbs tested (NIT B, NIT E, NIT F, NIT G and NIT H) and anti-GPIbα-sera-injected mice (Fig. 8b,c). As expected, DANA did not significantly increase platelet counts in anti-GPIIbIIIa mAb- or sera-injected mice (Fig. 8c; Supplementary Fig. 8). We then tested the therapeutic potential of oseltamivir phosphate (Tamiflu), a commonly administered anti-influenza medication that inhibits viral neuraminidase, and found significant amelioration of platelet counts in our murine model of anti-GPIbα-mediated ITP (Fig. 8d). In conclusion, this is the first study in mice to provide evidence to support utilization of sialidase inhibitors as a viable therapeutic alternative for refractory ITP patients.

## Discussion

We describe herein a novel FcγR-independent mechanism of platelet clearance in thrombocytopenia mediated predominantly by anti-GPIbα antibodies, which is distinct from Fc-dependent ITP in both mechanism and therapeutic management. We demonstrate both *in vitro* and *in vivo* that binding of mAbs to GPIbα induced significant platelet activation, surface NEU1 translocation and platelet desialylation. Importantly, we found that ITP patient plasma containing anti-GPIbα antibodies induced similar effects on healthy human platelets. This novel mechanism led to increased anti-GPIbα-mediated platelet sequestration in the liver and involvement of the hepatocyte in the phagocytosis of platelets in ITP via a previously undescribed pathway. Most importantly, our data demonstrate that sialidase inhibitors can block this pathologic process and ameliorate thrombocytopenia. These findings not only reveal a new mechanism of ITP, but also introduce a potential new diagnostic tool (for example, RCA-1 and NEU1 staining) and a novel therapeutic method (for example, sialidase inhibition), which will have a particularly significant impact on patients refractory to current ITP therapies.

The Fc-independent platelet clearance observed in anti-GPIbα-, but not anti-GPIIbIIIa-mediated thrombocytopenia, is most likely due to the distinct effects the antibodies have on their corresponding platelet receptors^17^,^18^. The ability of anti-GPIbα antibodies to affect platelet signalling has been previously described. We previously observed in murine models of fetal/neonatal alloimmune thrombocytopenia that anti-GPIbα antibodies increased both platelet activation and thrombus formation in the placenta resulting in miscarriage^25^. In addition, several studies have reported that anti-GPIbα antibodies affect platelet function independent of the Fc region of IgG. Specifically, F(ab)_2_ fragments of various anti-GPIbα antibodies induced platelet agglutination and GPIIbIIIa-dependent aggregation *in vitro*^30^,^47^ and caused thrombocytopenia *in vivo* when tested as anti-thrombotic agents^48^,^49^. Here, we found that anti-GPIbα antibodies induced platelet activation via an intracellular signalling cascade typical of von Willebrand factor binding. This led to the previously unobserved antibody-mediated desialylation of GPIbα. In contrast, anti-GPIIbIIIa antibodies did not induce these effects in murine platelets.

Interestingly, we found differences between murine and human platelet responses to anti-GPIIbIIIa antibodies, whereby anti-GPIIbIIIa mAbs and ITP patient plasma induced activation and desialylation of certain healthy donor platelets. We attributed this response to the low-affinity IgG receptor, FcγRIIa (the only FcγR expressed on human but not murine platelets), as IV.3 was shown to attenuate anti-GPIIbIIIa-mediated effects on human platelets. Therefore, it cannot be ruled out that some antibodies targeting GPIIbIIIa may also lead to platelet activation and desialylation, particularly in severe cases of ITP where high antibody titres may be sufficient to induce immune-complex binding via platelet FcγRIIa. Importantly, IV.3 had no significant effect on anti-GPIbα antibody-mediated human platelet activation and desialylation, suggesting that anti-GPIbα-mediated platelet effects predominantly follow F(ab)_2_ binding and are Fc independent. However, FcγRIIa may exacerbate the anti-platelet effects and explain the stronger anti-GPIbα-mediated responses seen in human versus mouse platelets. Through its tyrosine-based activation motif, FcγRIIa signalling has been previously shown to contribute to, and was correlated with antibody-mediated platelet activation^31^,^50^,^51^, particularly when associated with the GPIb complex^31^,^51^,^52^. This may also be a target mechanism for FcγR-dependent therapies, such as IVIG, in both anti-GPIIbIIIa and anti-GPIbα ITP patients who are responsive to the therapy^53^.

We thus show that the anti-platelet effects of anti-GPIbα are fundamentally different from those of anti-GPIIbIIIa, whereby anti-GPIbα antibodies in ITP crosslink adjacent GPIbα subunits causing GPIbα-receptor clustering and activation. While we found that desialylation is dependent on anti-GPIbα-induced platelet activation, we observed through sialidase inhibition assays that desialylation itself contributes to platelet activation. We propose that NEU1-mediated desialylation, through the removal of bulky sialic residues, facilitates further GPIbα clustering. As has been previously reported, GPIb complex clustering can induce platelet activation independent of GPIb agonist stimulation^41^,^44^. It is also conceivable that removal of bulky sialic residues may facilitate anti-GPIbα-antibody-mediated GPIbα crosslinking and signalling. These events synergize GPIbα signalling and platelet activation as demonstrated by increased P-selectin and NEU1 expressions. This indicates a positive feedback loop whereby platelet activation and NEU1-mediated desialylation reinforce each other, synergistically contributing to platelet clearance. This positive feedback loop may explain why relatively low levels of autoantibodies in ITP patients can cause severe thrombocytopenia.

To date, the prevailing view has been that platelet clearance in ITP is mediated by FcγRs of reticuloendothelial system macrophages, primarily in the spleen^2^. Here, we provide the first evidence of a new phagocytic process involving the hepatocyte in mediating destruction of antibody-opsonized platelets. Platelet clearance via the AMR is dominant in the absence of macrophages, which may be analogous to ITP patients undergoing typical first-line treatments. As hepatic sequestration of platelets in ITP patients is correlated with refractoriness to splenectomy^54^,^55^, our findings that the hepatocyte AMR plays a significant role in the clearance of anti-GPIbα-opsonized desialylated platelets provide a potential explanation for refractoriness to splenectomy, as well as to steroid and IVIG therapies.

The burden on hepatocytes in the clearance of antibody-bound desialylated platelets has never been studied. However, increased production of liver stress enzymes has been reported in untreated ITP patients^56^. Given that the liver is an important site for production of plasma coagulation factors required for hemostasis^57^, patients with anti-GPIbα antibodies may present with additional complications in the coagulation cascade and may experience a more severe bleeding phenotype. The liver is also the primary site for thrombopoietin production, thus there may be an indirect anti-GPIbα antibody effect on platelet production^46^. Long-term increased hepatic clearance of platelets may affect the coagulation system or decrease platelet production in anti-GPIbα-mediated ITP, and warrants further study.

We show in *FcγR*^*−/−*^ and *Aspgr2*^*−/*−^ mice that anti-GPIbα antibody-driven desialylation of platelet GPs significantly contributes to Fc-independent thrombocytopenia *in vivo*. This may explain findings in a recent report of a fatal case of refractory ITP where incubation of the patients' sera (positive for anti-GPIbα antibodies only) with normal healthy human platelets caused significant platelet activation and desialylation^58^.

We found anti-GPIbα-mediated platelet effects were susceptible to therapeutic intervention *in vivo*, as the sialidase inhibitors, DANA and oseltamivir, were able to rescue anti-GPIbα-mediated thrombocytopenia in mice. Clinical relevance of this finding is supported by a recent study in a small cohort of patients, which found a greater platelet increase in oseltamivir-administered individuals independent of influenza infection^59^. This follows a case report of the successful treatment of an anti-GPIbα-positive ITP patient with oseltamivir, who had been previously refractory to numerous first- and second-line therapies. Remarkably, on oseltamivir, he maintained a sustained platelet response^60^. It is possible that the therapeutic targets of oseltamivir in these patients may not only be limited to platelets, as sialidase inhibition may also target immune cells, contributing to the amelioration of thrombocytopenia through modulation of the immune response^61^,^62^. Conversely, NEU1 released from platelets may adversely target the same immune cells and amplify the anti-platelet response^63^. In addition, non-platelet-derived sialidases causing enhanced removal of desialylated platelets, as has been reported in various infectious diseases such as *Streptococcus pneumonia*^64^ and *Trypansoma cruzi* (Chagas' disease)^65^, may provide links with infections and thrombocytopenia. This may also occur with influenza infection^66^, although direct evidence is lacking to date.

These findings also provide insights into the platelet activation–desialylation dialogue and its positive feedback loop, which may contribute to thrombosis and cardiovascular diseases. Platelet activation and desialylation have been previously described in coronary heart disease (CHD) patients^67^,^68^, in which the sialic content of platelets was inversely correlated with the severity of CHD^67^. In addition, compared with patients without CHD, CHD patients overall exhibited more desialylated platelets that were prone to aggregation with higher sensitivity to weak agonists, such as ADP and serotonin^68^,^69^. These reports strongly reinforce the notion that platelet desialylation potentiates activation of platelets in other disease states, which may result in aggregation and platelet consumption. Thus, this novel Fc-independent platelet clearance pathway may also be involved in the clearance of activated platelets or platelet microaggregates including embolized thrombi, and is likely relevant to other thrombocytopenias caused by antibodies, such as fetal/neonatal alloimmune thrombocytopenia, post-transfusion purpura and drug-induced thrombocytopenias.

In summary, we have demonstrated that anti-GPIbα antibodies cause platelet activation and desialylation, two processes co-existing in a positive feedback loop. This leads to Fc-independent platelet clearance in the liver mediated by the AMR of the hepatocyte. Most importantly, this pathway is amenable to sialidase inhibition. Given that IVIG is extremely costly and a limited resource, and steroids are associated with severe side effects, these treatments should only be implemented to patients who will benefit. Patients with anti-GPIbα-mediated ITP who present with significant platelet desialylation may be identified as likely non-responders to conventional first-line treatments and splenectomy. We should also not exclude the potential impact of sialidase inhibitor therapy on anti-GPIIbIIIa-mediated ITP, as some anti-GPIIbIIIa antibodies may also cause platelet desialylation in human platelets. Clinical trials are required to test the diagnostic value of platelet desialylation and the therapeutic potential of sialidase inhibitors. Thus, our findings not only offer valuable implications for the diagnosis, prognosis and successful treatment of ITP, but also provide a potential mechanism in the clearance of activated platelets (emboli). As such, these findings may also have important implications in the field of alloimmune thrombocytopenias and cardiovascular diseases, although further investigation is required to test these hypotheses.

## Methods

### Mice

*GPIbα*^*−/−*^ mice and *GPIIIa*^*−/−*^ mice were originally described^25^ and provided by Dr Zaverio M. Ruggeri and Dr Jerry Ware (The Scripps Research Institute) and Dr Richard O. Hynes (Massachusetts Institute of Technology), respectively. The mice were backcrossed to BALB/c (Charles River Laboratories) background 10 times, then bred to generate syngeneic gene-deficient mice. *FcγR*^*−/−*^ (C.129P2(B6)-*Fcer1g*^*tm1Rav*^N12) (6–8 weeks) mice were purchased from Taconic. All aforementioned mice were housed in the St Michael's Hospital Research Vivarium, and all studies were approved by the St Michael's Hospital Animal Care Committee. Female mice between ages of 8 and 12 weeks were used for experiments. *Aspgr2*^*−/−*^ mice and related experiments were performed by Dr. Karin Hoffmeister's laboratory at the Harvard Medical School.

### Monoclonal antibody generation

*GPIIIa*^*−/−*^ or *GPIba*^*−/−*^ BALB/c female mice were transfused with 10^8^ WT washed platelets per week, for 4–6 weeks. Immunized splenocytes were then fused with mouse myeloma cells Ag8.653, and hybridomas were selected by hypoxanthine–aminopterin–thymidine medium. Positive hybridomas were identified using flow cytometry and subcloned by limiting dilution. Antibody-secreting hybridomas were cultured in sera-free medium for large-scale antibody production. Monoclonal antibodies were purified using protein-G sepharose beads.

### Platelet aggregation assay

Platelet aggregation was performed as previously described^28^,^26^,^27^,^70^. Platelet-rich plasma (PRP) was prepared from citrated whole blood (1:9 v/v) by centrifugation at 300*g* for 7 min. Gel-filtered platelets were purified from PRP using a Sepharose 2B chromatography column with PIPES buffer (5 mM PIPES, 137 mM NaCl, 4 mM KCl and 0.1% glucose, pH 7.0). Gel-filtered platelets or PRP (250 μl, 2.5 × 10^8^ ml^−1^) were incubated with PBS, control IgG or anti-GPIbα or anti-GPIIbIIIa mAbs for 10 min. To characterize anti-GPIIbIIIa antibodies, platelet aggregation was induced by 20 μM ADP (for PRP) or 1 U ml^−1^ thrombin (for gel-filtered platelets). As for anti-GPIbα antibodies, platelet aggregation in PRP was induced by 20 μg ml^−1^ botrocetin (for mouse) or 1.5 mg ml^−1^ ristocetin (for human) and monitored by a computerized Chrono-log aggregometer (Chrono-Log Corporation, Havertown, PA, USA).

### Patients

The patient plasma samples utilized in the present manuscript were from our previous published study of 176 ITP patients conducted at the Anhui Medical University Hospital, Hefei, China^16^. Informed consent was obtained from all subjects and the study was approved by the Anhui Medical University research ethics committee. Plasma samples were characterized with MAIPA, and only those samples highly specific with either anti-GPIbα with undetectable anti-GPIIbIIIa or anti-GPIIbIIIa with undetectable anti-GPIbα were selected for studies in this manuscript. Twenty-four untreated ITP patients' plasma samples were included: 12 with anti-GPIbα (patients ITP-1 to ITP-12) and 12 with anti-GPIIbIIIa (patients ITP-a to ITP-l) autoantibodies.

### Reagents and materials

Inhibitors were used for the following: intracellular Ca^2+^ increases: BAPTA-AM (EMD4 Biosciences), P38MAPK: SB203580 (Enzo Life Sciences), Src family protein tyrosine kinase: PP1 (Sigma), neuraminidase: *N*-acetyl-2,3-dehydro-2-deoxy neuraminic acid sodium salt (DANA) (EMD4 Biosciences) and oseltamivir phosphate (Santa Cruz), pan-caspases: Q-VD-OPh (Sigma), cAMP: prostacyclin (PGI_2_) (Cayman) and AMR: asialofetuin and its control fetuin (Sigma). OSGE (Cedarlane) was used to cleave GPIbα.

### Blood collection and platelet isolation

Procedures were approved by the Research Ethics Board of St Michael's Hospital (Toronto, ON, Canada) and conducted as previously described^25^,^26^,^58^. Venous blood was obtained from healthy volunteers by venipuncture into 3.2% trisodium citrate. PRP was prepared by centrifugation (10 min, 300*g*, no brake, 22 °C). Platelets were isolated from PRP and washed (15 min, 1,050*g*, no brake, 22 °C, with 10 ng ml^−1^ PGI_2_) and resuspended in HEPES Tyrode's solution (145 mM NaCl, 5 mM KCl, 0.5 mM Na_2_HPO_4_, 1 mM MgSO_4_, 5 mM HEPES and 5 mM glucose, pH 7.2). Platelets were counted with a Z2 Series Coulter Counter (Beckman Coulter), adjusted to 200 × 10^6^ platelets per ml and stored at 22 °C for 30 min to regain responsiveness. Sera were isolated from blood of healthy donors by high-speed centrifugation of the platelet-poor plasma and red blood cell fraction (5 min, 2,000*g*, 22 °C). For isolation of murine platelets, blood was collected from anesthetized mice in 100 μl of 130 mM trisodium citrate by retro-orbital eye bleed, and washed platelets were prepared as described above with human-washed platelets.

### *In vitro* platelet–antibody assays

Platelets were treated with various mAbs (2.5 μg ml^−1^) or with sera from *GPIb*^*−/−*^ or *GPIIIa*^*−/−*^ mice that have been intravenously immunized 4 × weekly with 10^8^ WT platelets^25^. Human platelets were also treated with sera from ITP patients (1/50 v/v) in HEPES Tyrode's solution, for 60 min at 22 °C. Unbound antibody was removed by centrifugation (15 min, 1,050*g*, no brake, 22 °C, with 10 ng ml^−1^ PGI_2_). Platelets were resuspended in Tyrode's solution (pH 7.2) and incubated (30 min, 22 °C) to restore responsiveness before measurements. For visualization of antibody-mediated platelet aggregate formation, human or murine PRP were incubated with various mAb (2.5 μg ml^−1^) under stirring conditions (1,000 r.p.m.) for 10 min. The images of platelet aggregates were recorded using a digital camera (DP70; Olympus, Tokyo, Japan) under a Zeiss Axiovert 135 inverted fluorescent microscope (Zeiss, Oberkochen, Germany). In some experiments, various inhibitors were used, prior to addition of mAbs (15 min, 22 °C), P38MAPK inhibitor SB203580 (10 μM), Ca^2+^ quencher BAPTA-AM (20 μM), Src family protein tyrosine kinase inhibitor PP1 (10 μM), Q-VD-OPh (50 μM), neuraminidase inhibitor DANA (1 mM) or FcγRIIa/III inhibitor IV.3 (StemCell Technologies) (2.5 μg ml^−1^). In some experiments OSGE (80 μg ml^−1^) was used to remove the extracellular part of GPIbα following antibody incubation, as previously described^43^.

### Flow cytometry

P-selectin expression was detected with FITC-labelled anti-P-selectin antibody (5 μg ml^−1^) (BD Biosciences) for murine platelets, or with PE-labelled (2 μg ml^−1^) (eBioscience) or Brilliant Violet 605 (0.5 μg ml^−1^) (Biolegend) anti-human CD62P (P-selectin) antibody for human platelets. GPIIbIIIa activation was detected with JON/A-PE (5 μl per 10^6^ platelets) (Emfret) or PAC-1-FITC (20 μl per test) (BD Biosciences) following platelet fixation in 1% paraformaldehyde for 15 min on murine and human platelets, respectively. To quantify glycosylation, platelets were incubated with fluorescein-labelled RCA-1 or fluorescein-labelled PNA or biotinylated Maackia Amurensis Lectin II or fluorescein-labelled Sambucus Nigra Lectin. All were purchased from Vector Laboratories and used at 0.5 μg ml^−1^, 15 min, 22 °C to measure exposure of sugar residues. Surface NEU1 expression was measured with rabbit anti-NEU1 antibody (clone H-300 Santa Cruz, 1:50 dilution) and detected with Alexa Fluor 647 secondary antibody (0.8 μg ml^−1^) (Molecular Probes/Invitrogen). Murine IgG binding was detected with Alexa Fluor 647 goat anti-mouse IgG (H+L) (0.8 μg ml^−1^) (Invitrogen) or. Fold increase is represented as percentage increase from gated control platelets set at 1% positive. A total of 10,000 platelet events were acquired and analysed on a MACSQuant (Miltenyi Biotech).

### Western blotting

Human platelets were incubated with mAb (NIT A, NIT B or NIT F) and DANA or 10 μM PP1, or 10 μM SB253580 as described above and lysed in 1 × Laemmli buffer. For detection of phospho-p38MAPK, 1 × Halt protease inhibitor cocktail (Thermo Scientific) was also added. Samples were reduced with addition of 0.1 M dithiothreitol and boiled at 95^°^C for 10 min. Platelet lysate was separated by SDS–polyacrylamide gel electrophoresis, transferred onto polyvinylidene difluoride membrane (GE Healthcare). For RCA-1 staining, membranes were probed with biotin-conjugated RCA-1 (2 μg ml^−1^) (Vector Labs) according to the manufacturer's instructions, mouse anti-human CD41b antibody (1:5,000) (clone EPR6995, Abcam) and with β-actin antibody (1:400) (clone C1, Santa Cruz). For phospho-p38MAPK, membranes were probed with phospho-p38MAPK (Thr180/Tyr182) antibody (1:5,000) (Cell Signaling Techonology) at 4 °C overnight. Avidin conjugation to RCA-1 was performed with a Vectastain ABC kit (Vector Labs) according to the manufacturer's instructions. HRP-conjugated secondary anti-mouse antibody (1:5,000) (Santa Cruz) was then utilized. Blots were developed by ECL (Thermo Scientific) and visualized on the VersaDoc MP 4000 imaging system (Bio-Rad). Uncropped immunoblots are shown in Supplementary Fig. 9.

### Antibody fab generation

NIT A, NIT B and NIT F Fab fragments were generated with Thermo Scientific IgG1 F(Ab)_2_ and Fab preparation kits according to manufacturers' protocol. Briefly, 1 mg ml^−1^ of antibody was desalted and digested with immobilized Ficin (beaded agarose resin) in the presence of 25 mM cysteine. Digest products were purified with Protein-G beads and dialysed and concentrated with Amicon Ultra-4 Centrifugal Filter Units (Millipore). Purified Fab was run on 10% SDS–polyacrylamide gel for visualization.

### Immunocytochemistry

Platelets treated with anti-GPIbα or anti-GPIIbIIIa antibodies as described above were fixed in 4% paraformaldehyde at 22 °C for 20 min and spun down onto poly-L-lysine-coated coverslips (BD Biosciences; 500*g*, 5 min). Cells were blocked overnight in PBS with 1% BSA at 4 °C. Cells were incubated with primary antibodies rabbit anti-NEU1 IgG (4 μg ml^−1^) (Santa Cruz) and our anti-CD61 mAb M1 (5 μg ml^−1^) overnight at 4 °C. The platelets were washed in triplicate with PBS before addition of species appropriate secondary antibodies Alexa Fluor 488 and 647 (1:10,000) (Invitrogen). Images were taken with a Zeiss LSM 700 Confocal laser scanning microscope with a × 63 objective. Images were analysed with ZEN 2010 software (Zeiss) and Adobe Photoshop 7.

### *In vivo* platelet activation and desialylation

The passive murine ITP model was used to induce thrombocytopenia^12^. Various anti-platelet mAbs were injected intraperitoneally. The amount of mAbs used was determined following titration to induce similar levels of thrombocytopenia. After titration, 0.75 μg of anti-GPIbα mAb (0.03 μg g^−1^ total body weight) was found to be comparable to injection of 4 μg of anti-GPIIbIIIa mAb (0.16 μg g^−1^ total body weight). In some experiments, neuraminidase inhibitor DANA (2 mg or 0.08 mg g^−1^ total body weight) was injected intraperitoneally 2 min prior to injection of mAbs. Alternatively, oseltamivir phosphate (0.25 mg or 0.01 mg g^−1^ total body weight) was therapeutically intravenously injected 2 h after antibody injection. After 16 h, mice were bled via the saphenous vein, platelet count was measured with Coulter Z counter and PRP was analysed for P-selectin and RCA-1 binding via flow cytometry as described above.

### *In vivo* platelet clearance

A total of 10^8^ washed murine platelets were prepared as described above and labelled with 5 μM of CMFDA and incubated with anti-platelet mAbs (5 μg ml^−1^, 1 h, 22 °C), washed and injected intravenously into WT BALB/c mice. Platelet circulation was measured after transfusion of CMFDA-labelled platelets via enumeration of fluorescently labelled platelets in whole-blood samples after 1 (baseline), 15 and 30 min. To assess the contribution of AMRs on hepatocytes, a bolus injection of inhibitor asialofetuin (0.2 mg g^−1^ total body weight) was administered via the tail vein and fetuin was used as control, as previously described^24^. At 10 min post transfusion, a booster injection of asialofetuin (0.1 mg g^−1^ total body weight) was administered intraperitoneally. Some mice were depleted of their macrophages via intravenous injection of Clondrate-encapsulated liposomes (0.01 ml g^−1^ body weight) 48 h prior.

### Immunohistochemistry

Following *in vivo* platelet clearance studies as described above, mice were anesthetized and drained of their blood via retro-orbital bleeding. Liver and spleen were harvested and snap-frozen in liquid nitrogen. Frozen tissue was sectioned (5 μm) with Leica Cryostat and fixed onto slides in ice-cold methanol. Slides were washed in 2% BSA and then incubated with primary rat anti-mouse F4/80 (1:1,000) (clone BM8, eBioscience) or rabbit anti-ASPGR1/1 (1:1,000) (Santa Cruz) overnight at 4 °C. Slides were then stained or anti-rat Cy3 or anti-rabbit Alexa Fluor 647 secondary antibody (1:5,000) for 2 h. Sections were mounted with Vectashield mounting medium containing 4′,6-diamidino-2-phenylindole. Images were captured using Olympus upright fluorescence microscope and analysed with Adobe Photoshop 7 and Image J.

### Statistical analysis

All data are presented as mean±s.e.m. unless otherwise indicated. Statistically significant differences between controls were assessed by the Student's unpaired *t*-test (two tailed). Statistically significant differences between multiple groups were assessed by one-way or two-way analysis of variance with Bonferroni *post hoc* analysis, as indicated. Statistical analyses were performed using Prism software (GraphPad). A *P* value of 0.05 or less was considered significant.

## Additional information

**How to cite this article:** Li, J. *et al*. Desialylation is a mechanism of Fc-independent platelet clearance and a therapeutic target in immune thrombocytopenia. *Nat. Commun*. 6:7737 doi: 10.1038/ncomms8737 (2015).

## Supplementary Material

Supplementary Information Supplementary Figures 1-9 and Supplementary Table 1

## Figures and Tables

**Table 1:** Characterization of mouse anti-mouse mAb against GPIbα and GPIIIa.

Clone name	IgG subtype	Antigen	Species cross-reactivity (tested)	Inhibits murine platelet aggregation	Inhibits human platelet aggregation
**NIT A**	**IgG 2a**	**GPIbα**	**h, m, p**	**Yes**	**Yes**
**NIT B**	**IgG 2a**	**GPIbα**	**h, m, p**	**Yes**	**Yes**
**NIT F**	**IgG 2a**	**GPIbα**	**h, m, p**	**Yes**	**Yes**
					
NIT E	IgG 2b	GPIbα	m	Yes	NA
NIT G	IgG 1	GPIbα	m	Yes	NA
NIT H	IgG 1	GPIbα	m	Yes	NA
**9D2**	**IgG 1**	**GPIIbIIIa**	**h, m, p, r**	**Yes**	**No**
**M1**	**IgG 1**	**GPIIbIIIa**	**h, m, p, r**	**Yes**	**Yes**
**PSI B1**	**IgG 1**	**GPIIIa (PSI domain)**	**h, m, p, rib**	**Yes**	**Yes**
**PSI C1**	**IgG 1**	**GPIIIa (PSI domain)**	**h, m, p, rib**	**Yes**	**Yes**
**PSI E1**	**IgG 2b**	**GPIIIa (PSI domain)**	**h, m, p, rib**	**Yes**	**Yes**
**HUTA A**	**IgG 2a**	**GPIIbIIIa**	**h**	**NA**	**Yes**
**HUTA B**	**IgG 1**	**GPIIbIIIa**	**h, m, p**	**No**	**Yes**

IgG, immunoglobulin G; mAb, monoclonal antibody; NA, not applicable.

mAbs were generated in *GPIbα*^*−/−*^ and *GPIIIa*^*−/−*^ mice. In addition to mouse (m), antibodies were also cross-reactive to other species, including tested human (h), pig (p), rat (r) and rabbit (rib). Antibodies cross-reactive with human antigens are in bold. ADP (20 μM)- or thrombin (1 U)-induced human/mouse platelet aggregation was inhibited by most anti-GPIIbIIIa mAbs. Ristocetin (20 μg ml^−1^)-/botrocetin (1.5 mg ml^−1^)-induced human/mouse platelet aggregation was inhibited by anti-GPIbα mAbs.
