# Supplementary material for: Desialylation is a mechanism of Fc-independent platelet clearance and a therapeutic target in immune thrombocytopenia
Source: Nat Commun. 2015 Jul 17;6:7737. doi: 10.1038/ncomms8737 (PMC4518313; doi:10.1038/ncomms8737)
Supplement: Supplementary Information — Supplementary Figures 1-9 and Supplementary Table 1 [file ncomms8737-s1.pdf]

Supplementary Figure 1

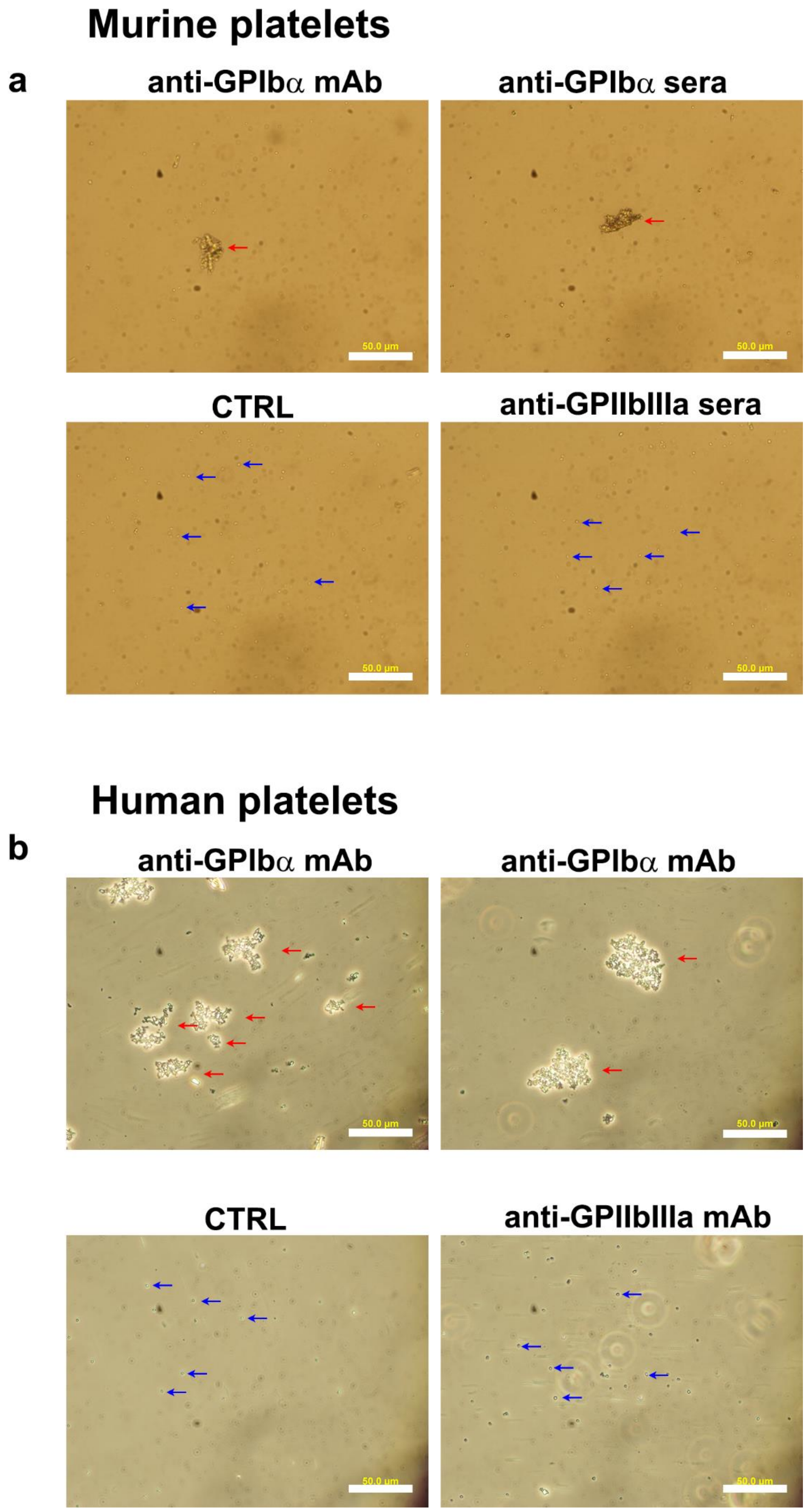

Supplementary Figure 1. Anti-GPIbα, but not anti-GPIIbIIIa antibodies induces platelet aggregation. (**a,b)** Representative images of platelet aggregates visualized by light microscopy following incubations with anti-GPIbα (NIT G (murine), NIT B and NIT F (human)) or anti-GPIIbIIIa (PSI C1 (murine) and HUTA A (human)) mAb or sera (murine). Control platelets (CTRL) were incubated with non-specific murine IgG or IVIG (human). *n*=3-4. Red arrows point to representative platelet aggregates. Blue arrow point to single non-aggregated platelets. Scale bars represent 50μM.

Supplementary Figure 2

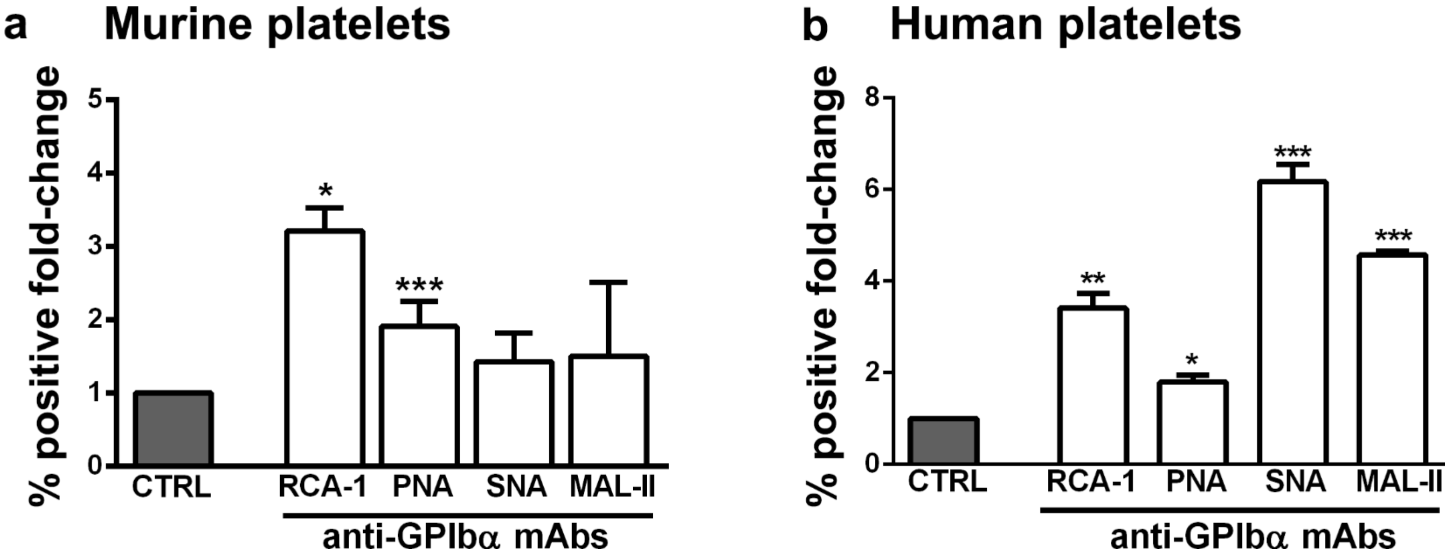

Supplementary Figure 2. Glycosylation profile as assessed by flow cytometry of (**a**) murine and (**b**) human platelets following incubation with anti-GPIbα mAbs. Exposed galactose was detected with Ricinus Communuis Agglutinin (RCA-1), Gal-β(1,3)GalNAc was detected with Peanut Agglutinin (PNA), sialic acids in α2,6 and α2,3 linkages were detected with Sambucus Nigra Lectin (SNA) and Maackia Amurensis Lectin II (MAL II) respectively. Although only significantly increased binding of PNA was observed on murine platelets, increased binding of all the lectins to human platelets occurred following anti-GPIbα mAbs incubations. We postulate additional sialic acids may originate from released platelet contents, such as VWF or P-selectin, following antibody mediated activation, netting in an overall lack of significant change or increase in surface sialic content. *n*=6. Anti-GPIbα mAbs shown as mean ± SEM of individual mAbs. **P*<0.05, ***P*<0.01, ****P*<0.001 vs. CTRL as determined by Student's *t*-test.

Supplementary Figure 3

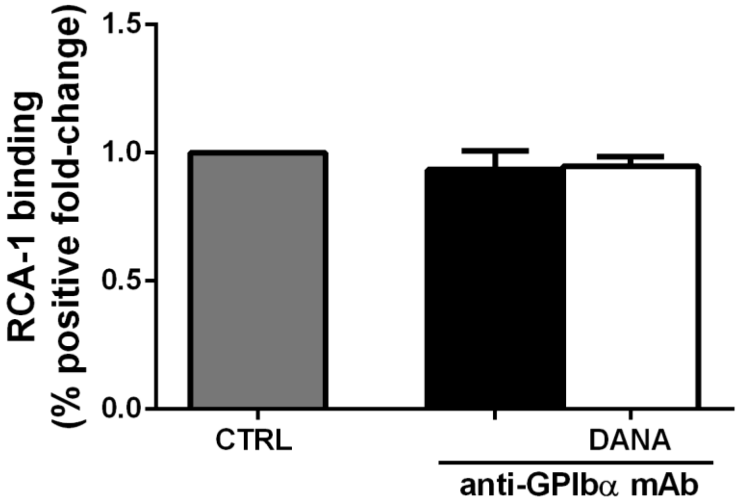

Supplementary Figure 3. No significant change in RCA-1 binding on *GPIbα* $^{-/-}$ platelets. Washed *GPIbα* $^{-/-}$ platelets were incubated with various GPIbα mAbs (NIT A, NIT B, NIT E, NIT F, and NIT G) as with WT platelets. *n*=2-3 per mAb. Data is expressed as fold change from non-specific murine IgG treated control platelets (CTRL) and as mean ± SEM of individual mAbs.

Supplementary Figure 4

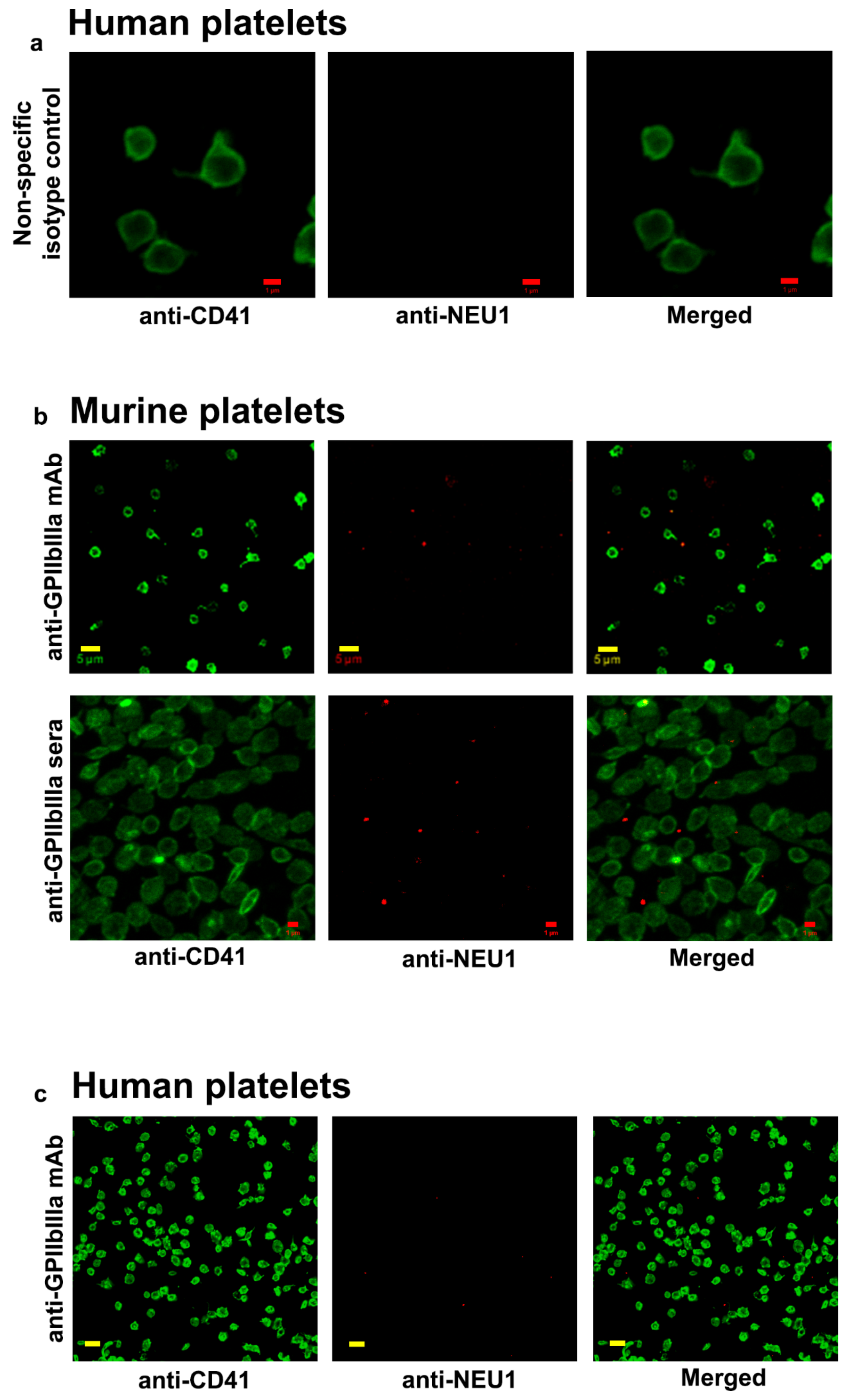

Supplementary Figure 4. Anti-GPIIbIIIa antibodies does not cause platelet NEU1 surface expression (**a)** Confocal images of human platelets and isotype control (non-specific rabbit polyclonal IgG). (**b,c)** Representative confocal images of surface expression of NEU1 on murine (**a)** and human (**c)** platelets stained with anti-NEU1 and anti-CD41 following incubations with anti-GPIIbIIIa mAb (9D2 (murine), PSI C1 (human)) or anti-GPIIbIIa sera (murine). NEU1 positivity similar to control sera treated platelets (Fig. 2a), and does not appear to co-localize with platelet surface marker CD41. All other mAbs were also tested with similar results. *n*=5-8. Red scale bars are 1μM, yellow scale bars are 5μM.

Supplementary Figure 5

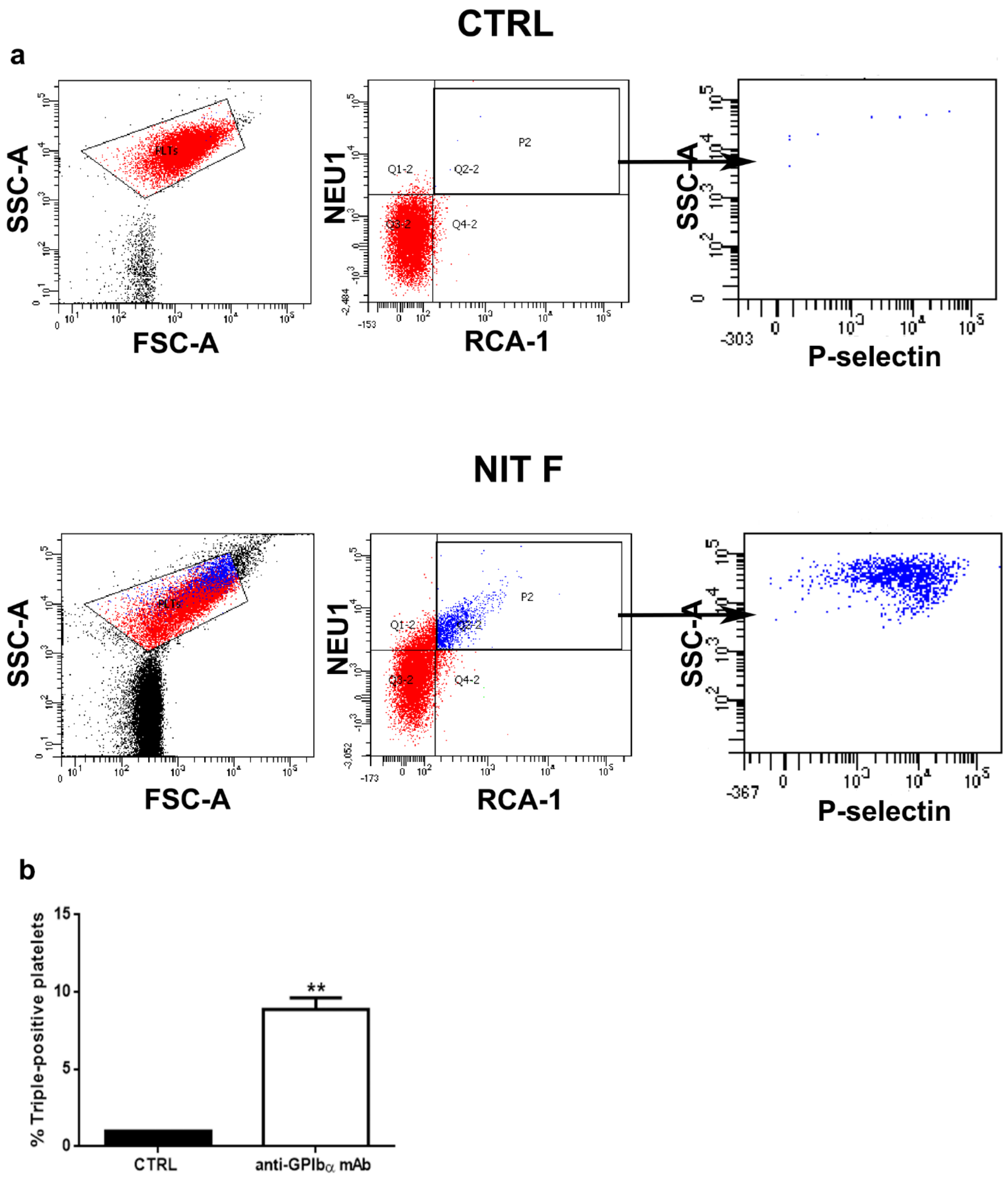

Supplementary Figure 5. Significant increase of triple positive P-selectin, RCA-1 and NEU1 human platelets following anti-GPIbα mAb incubation. (**a**) Representative dot-plots of triple stained RCA-1, NEU1 and P-selectin of CTRL (IVIG) and NIT F (anti-GPIbα mAb) treated platelets. Gated washed platelets (red) double positive for NEU1 and RCA-1 (blue) were gated (P2) and plotted on P-selectin (last panel). (**b**) Graph of total percentage of triple positive platelets. Anti-GPIbα mAb shown as mean ± SEM of individual mAbs. N=3. ***P*<0.01 vs. CTRL as assessed by Student's *t*-Test.

Supplementary Figure 6

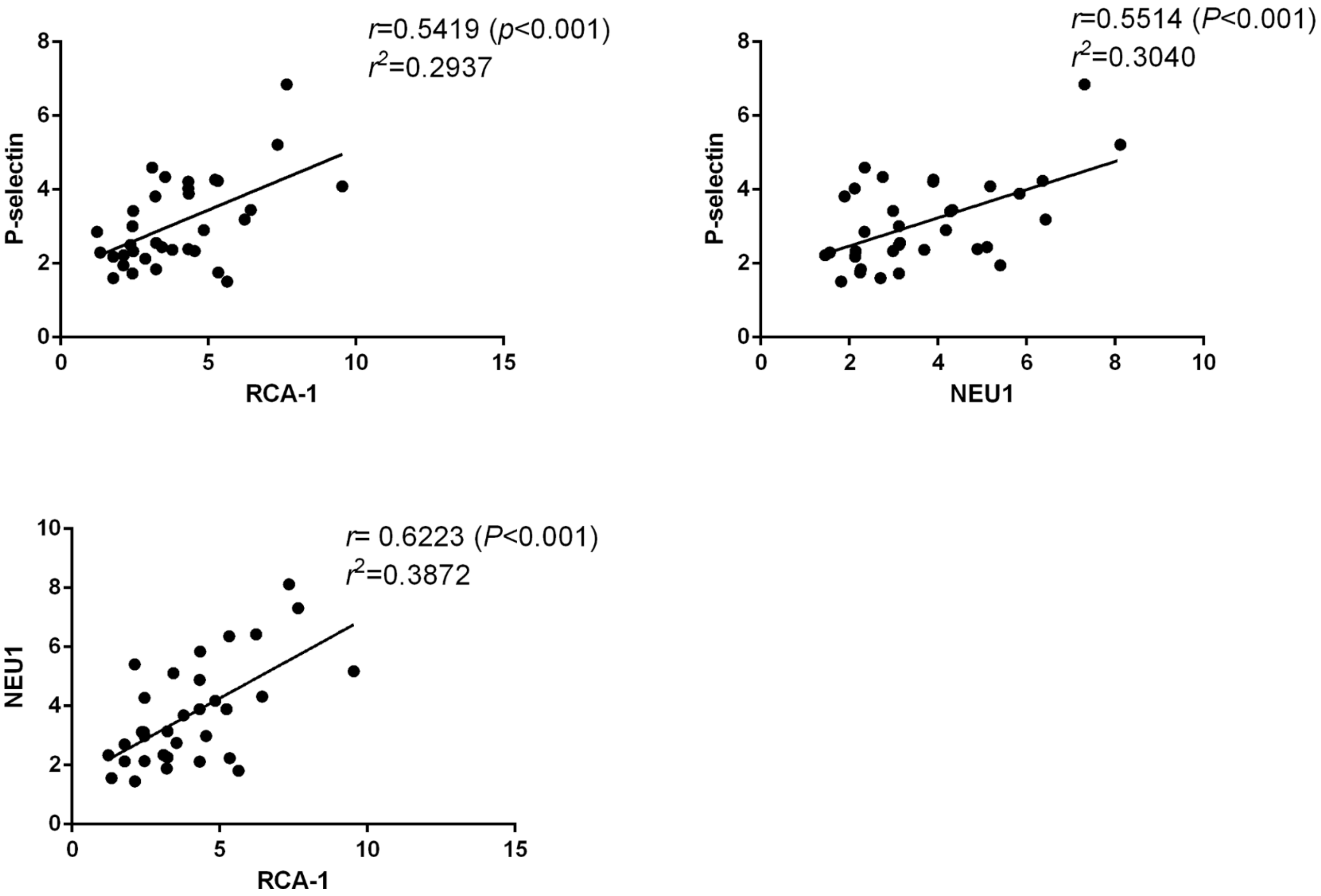

Supplementary Figure 6 Significant positive correlation between anti-GPIbα mAb mediated P-selectin and NEU1 expression and RCA-1 binding in murine platelets as assessed by Pearson Correlation Coefficient (*r*). Each dot represents one sampling from one individual mAb. The straight-line represents the best-fit line obtained by linear regression analysis.

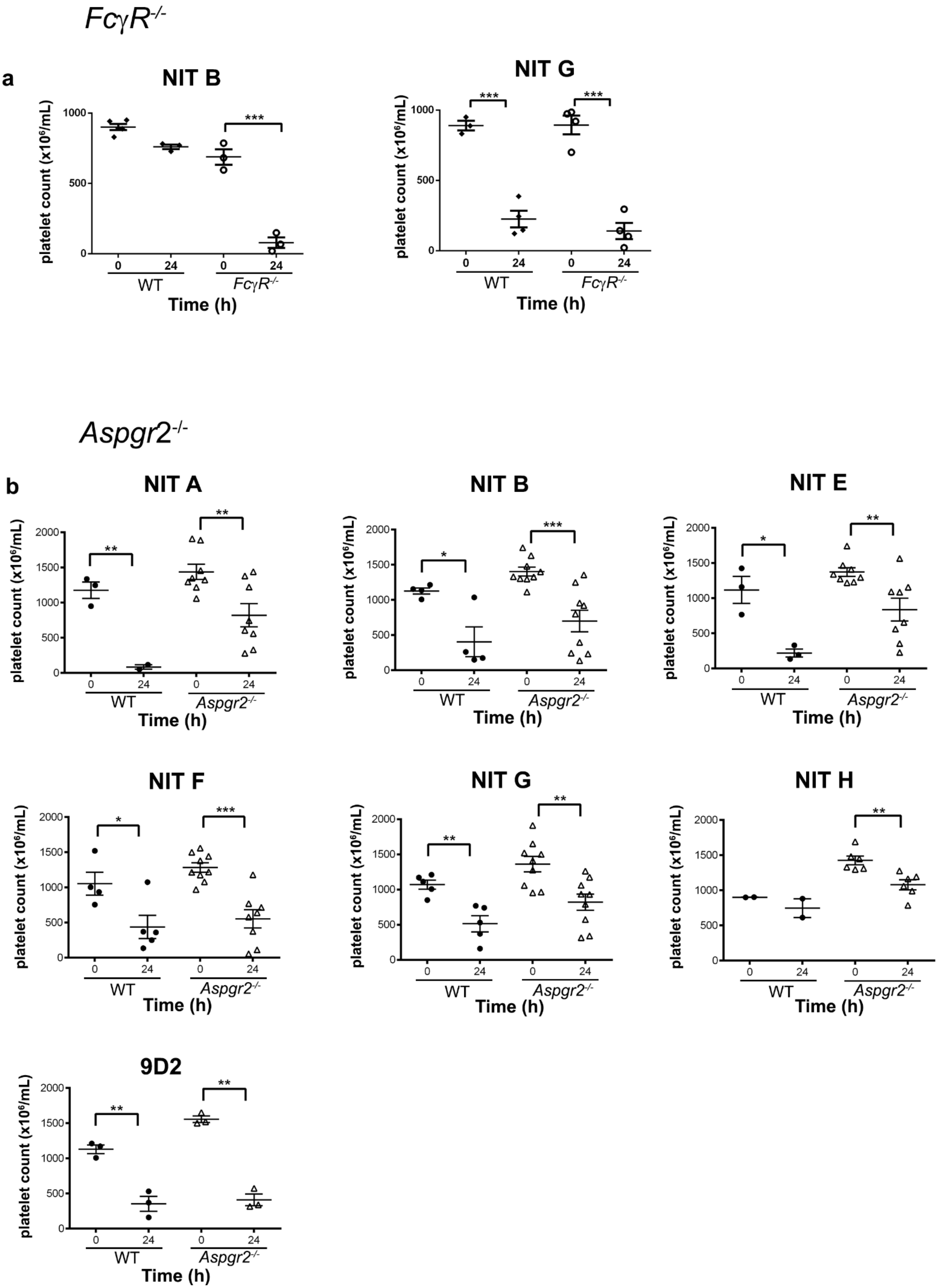

Supplementary Figure 7

Supplementary Figure 7: (a) Anti-GPIbα mAb induces thrombocytopenia in *FcγR*$^{-/-}$ mice. Indicated mAbs were injected into WT Balb/C or *FcγR*$^{-/-}$ at the same dose (b) Anti-GPIbα but not anti-GPIIbIIIa mAb mediated thrombocytopenia is less severe in *Aspgr2*$^{-/-}$ mice compared with WT control. Indicated mAbs were injected into WT C75/BL6 or *Aspgr2*$^{-/-}$ at the same dose. Platelet enumeration was done immediately prior to mAb injection (time 0) and 24 hours following (time 24). *P<0.05, **P<0.01, ***P<0.001 as determined by Student's *t*-test.

Supplementary Figure 8

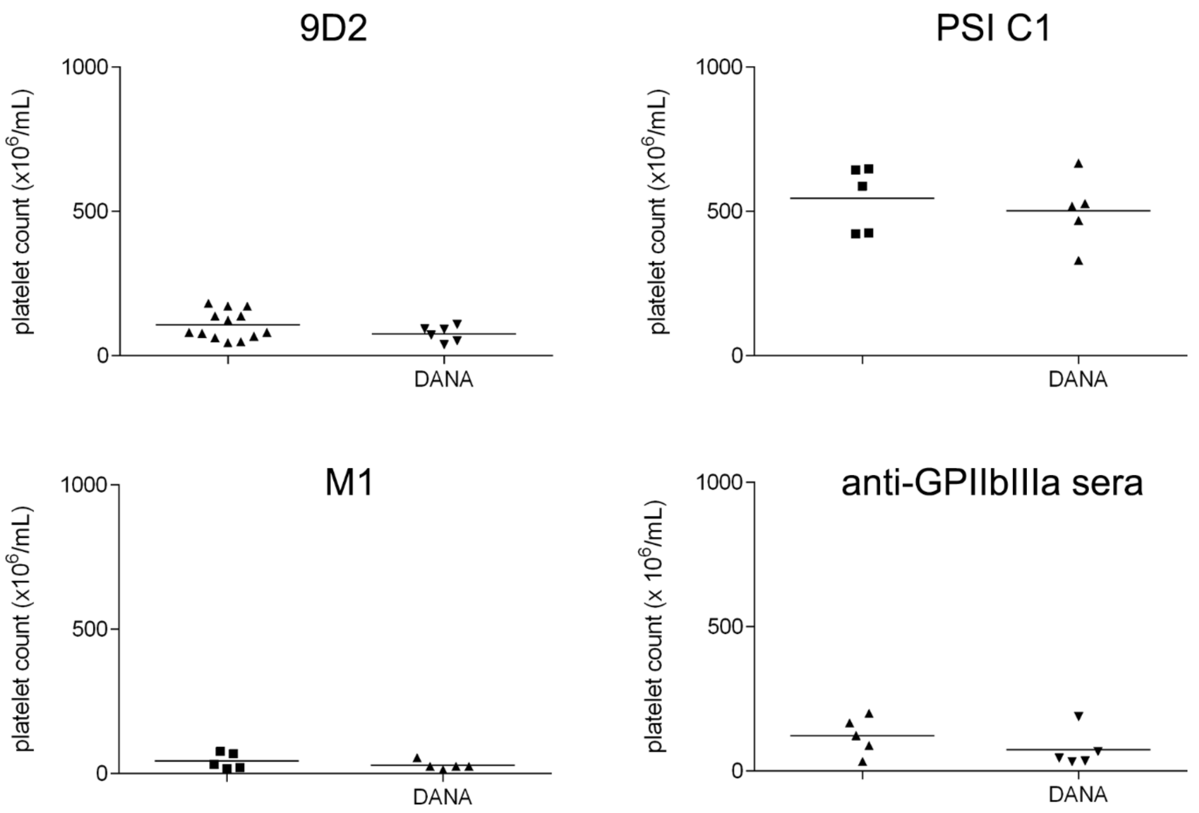

Supplementary Figure 8. DANA does not rescue anti-GPIIbIIIa mediated thrombocytopenia. DANA or PBS was injected immediately prior to anti-GPIIbIIIa mAb of sera injection to induce thrombocytopenia. Platelet numbers were enumerated and compared between DANA treated or mock (PBS) treated groups.

Supplementary Figure 9

Figure 2k

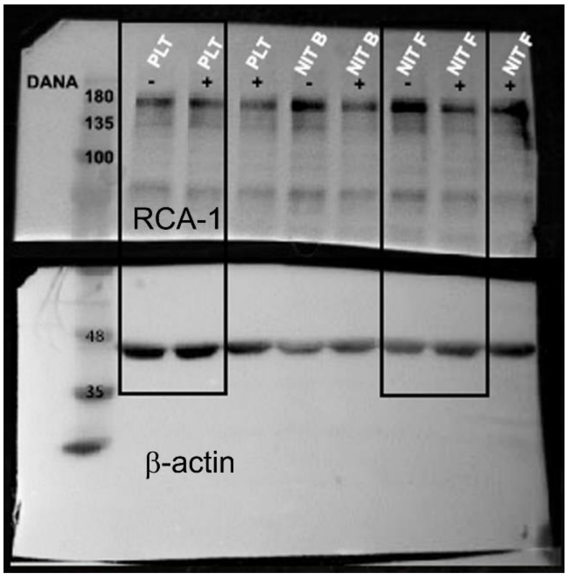

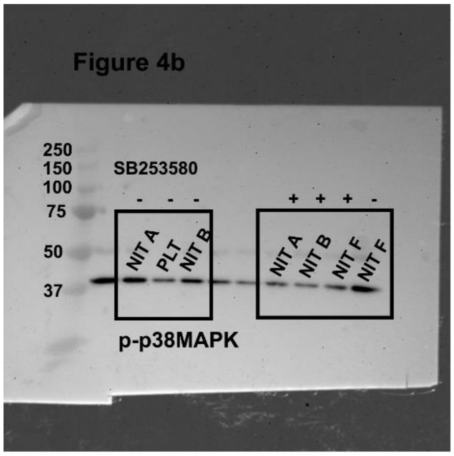

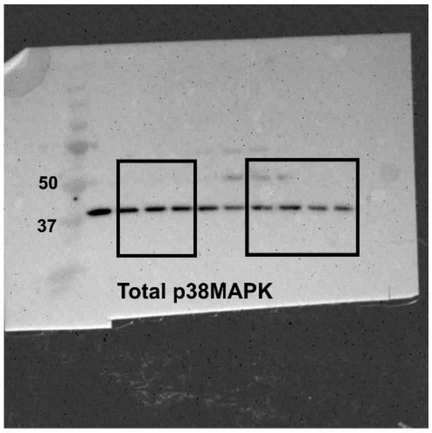

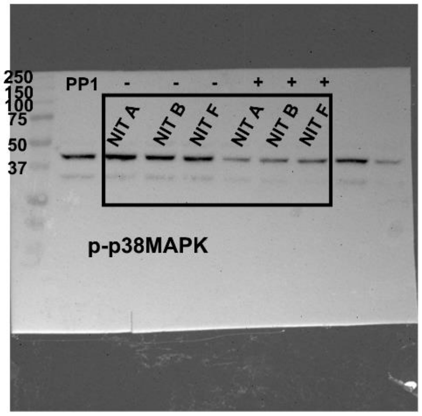

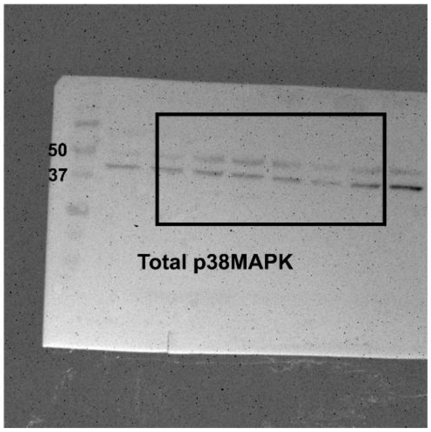

Supplementary Figure 9. Uncut immunoblots of Figures 2k, and 4b.

Supplementary Table 1

Injections	PBS	Anti-GPIbα mAb	Anti-GPIIbIIIa mAb
RBC (x10^9/L)	8.095±0.2245	7.951±0.3972	8.106±0.3390
WBC(x10^9/L)	8.599±1.089	9.354±1.251	8.738±0.9821
PLT(x10^6/ml)	790±129.5	46.88±33.25***	74.52±60.04***

Complete blood counts of WT Balb/c mice 24 hours post-injection of anti-GPIbα mAbs (1 μg) or anti-GPIIbIIIa mAbs (4 μg) or PBS. Peripheral red blood cell counts (RBC) and platelets (PLT) were determined with a HEMAVET HV950FS (Drew Scientific) counter. White blood cells (WBC) was counted on a hemacytometer following Ammonium-Chloride-Potassium (ACK) buffer RBC lysis. Significant decrease in platelet counts (PLT), with no significant changes in RBC or WBC counts in anti-GPIbα and anti-GPIIbIIIa mAb injected mice compared with PBS. N=4-11 in each group. Anti-GPIbα and anti-GPIIbIIIa mAb shown as mean ± SD of individual mAb. ****P*<0.001 vs. PBS as assessed by Student's *t*-Test.
